# Supplementary material for: What are the features of high-performing quality improvement collaboratives? A qualitative case study of a state-wide collaboratives programme
Source: BMJ Open. 2023 Dec 13;13(12):e076648. doi: 10.1136/bmjopen-2023-076648 (PMC10729078; doi:10.1136/bmjopen-2023-076648)
Supplement: Supplementary data [file bmjopen-2023-076648supp002.pdf]

A qualitative study of the Michigan collaborative quality initiatives

Interview topic guide

20.08.2017

Study aims

1. Produce a thorough empirical description of what it is that the Michigan CQIs do: the activities they undertake, the structures through which they operate, and the resources they use, for example.
2. Identify the program theory of the Michigan CQIs, including identification of specific interventions and their contextual influences, in so doing characterising the mechanisms of their success.
3. Generate learning about the distinctive features of the Michigan approach for generalisability and reproduction of improvement in other settings.

Interviewees

This document presents a topic guide for interviews with personnel who have participated or are actively participating in one or more of the Michigan collaborative quality initiatives (CQIs). Relevant personnel may include (but are not limited to):

- Senior leaders from Blue Cross Blue Shield of Michigan.
- Leaders of the CQIs.
- Program managers and implementers of CQI quality improvement programs.
- Front line staff from Michigan hospitals who participate in the CQIs.

Questions used may vary by role of personnel in the CQI, i.e. not all questions may be relevant for every interviewee.

Topic structure

1. Questions about role and relevant CQI(s).
2. Questions to elicit empirical description.

A qualitative study of the Michigan collaborative quality initiatives

Interview topic guide

20.08.2017

3. Questions to identify program theory.
4. Questions to identify features of context.
5. Questions about generalizability.
6. Safety net.

1. Questions about role and relevant CQI

1. Which CQI(s) were you involved in the past, or are you involved in now?

2. What was/is your role in the CQI(s)?

3. Please tell me about your role in the CQI(s).

2. Questions to elicit empirical description

Aim: Produce a thorough empirical description of what it is that the Michigan CQIs do: the activities they undertake, the structures through which they operate, and the resources they use, for example.

Structures, processes and activities

4. Please describe how the collaborative works – how is it structured, and what are its key processes and activities?

5. How do you ensure key processes and activities happen effectively and in standardized or harmonized way across different sites?

Resources

6. What resources does the collaborative need to operate? Which resources are particularly important, and why?

A qualitative study of the Michigan collaborative quality initiatives

Interview topic guide

20.08.2017

Topic selection and prioritization

7. How are topics or problems for quality improvement selected and prioritized? How are these decisions taken and by whom? How far is there consensus on what is important?

Definition and identification of positive deviance

8. How are high performing hospitals defined and identified? Are you most interested in hospital, unit or individual clinician performance? Why?

Technical components and interventions

9. What are the technical components, e.g. information technology and communications infrastructure, needed for improvement to happen? What kind of testing did you do to figure out which components were essential? Were there any challenges in making sure all components worked reliably at different sites?

10. How does technology enable improvement in Michigan? How is new technology selected and tested?

11. When variation in quality or practice is identified, how are best practices at the high performing hospitals identified?

12. Among the identified ‘best practices’ of high performing hospitals, how are the most important interventions to improve quality at other hospitals identified and selected?

13. How are these best practices developed and tested as quality improvement interventions, and how are they then introduced at other sites? What are the important processes and activities involved in this? How does it happen in practice?

A qualitative study of the Michigan collaborative quality initiatives

Interview topic guide

20.08.2017

Technical and operational expertise

14. What technical expertise is required for the collaborative to operate effectively? Is training required? Who receives training and how does that happen?

15. Who are the key people responsible for ensuring the collaborative works effectively on a day-to-day basis? Are the systems they use to achieve this reliable? What are they?

Data

16. What data are most relevant for the purposes of improving quality of care? Why? What process was used to prioritise data and what role did clinicians play in this?

17. How has data and its collection been standardized between participating hospitals? How did that process happen in practice?

18. How are data collected in Michigan?

19. Who is responsible for collecting data? What role do participating hospitals, units and clinicians have in the collection and preparation of data?

20. Please tell me about the data infrastructure used in Michigan. How does it provide what you need to improve quality? What are its most important features?

21. Was the data infrastructure planned and tested before the collaborative launched? Did you have a clear idea about what you needed in advance? How has it changed over time and why did it change?

A qualitative study of the Michigan collaborative quality initiatives

Interview topic guide

20.08.2017

22. How does the collaborative assure the quality of its data?

23. How much credibility does data have among participants? Where does that credibility come from? Why do participants trust the data?

24. Please tell me about how data is used to improve quality in Michigan. What are the key processes involved?

25. In your experience, what is unique or different about how data is collected and used in Michigan?

26. Is data used to rank or compare participants in any way? Does this happen at the level of hospitals, units or individuals? Can sites or individuals compare their performance?

Feedback

27. What information is fed back to participants? What aspects of quality or performance are highlighted as most important and why, e.g. are outcomes more important than process measures? How was this determined and what role did clinicians play?

28. How is information fed back to participants? What are the key processes and activities? To whom and how frequently does feedback occur? At what levels are information presented, e.g. hospital, unit, individual?

29. In what format is information fed back, e.g. use of visuals, text, numerical? How was this format developed? Who was involved in the process of developing this format? What role did clinicians play in developing it?

30. To what extent is information customised for individuals? Does the information presented vary by type of personnel, e.g. between doctors and nurses?

A qualitative study of the Michigan collaborative quality initiatives

Interview topic guide

20.08.2017

31. How is information fed back in a way that makes it actionable for improvement? How and at what level are actions identified, e.g. for hospitals or individual clinicians? How are action plans developed?

32. Is the approach to feedback in Michigan based on any particular theories? Was a particular approach to feedback planned from the beginning?

33. In your experience, what is unique or different about how feedback happens in Michigan compared to elsewhere?

Communication

34. What are the important communication principles and processes? What makes communication in Michigan effective?

35. Is there direct communication between participants in the collaborative? How does this happen in practice?

Research outputs

36. Why are the collaboratives able to publish so many of their results as research papers? What infrastructure and relationships are in place to support this approach? How does it happen in practice?

3. Questions to identify program theory

Aim: Identify the program theory of the Michigan CQIs, including identification of specific interventions and their contextual influences, in so doing characterising the mechanisms of their success.

A qualitative study of the Michigan collaborative quality initiatives

Interview topic guide

20.08.2017

Aims and development

37. Can you tell me what your original aim was? What problem was the collaborative created to solve? Did the aim change over time?

38. In the beginning, how was it imagined that the collaborative would achieve that aim? What structures and processes were assumed to be important, and why? By what mechanisms were these intended to improve quality?

39. Can you tell me how the collaborative was originally developed, and why it was developed in that way? What was the role of research evidence?

40. Over time, did the way you sought to improve quality change? What changed and why?

Key interventions for improvement

41. Can you give some examples of important interventions that make improvement happen in Michigan? How do they work?

42. Do any of these interventions work differently in practice to what was anticipated? Are some interventions more important or less important than you anticipated?

43. Are there any structures, processes or activities that developed and became important for improvement, that were not initially imagined or planned?

44. In your experience, what advantages does the Michigan approach have over other methods improving quality? What is different about what happens in Michigan that makes you better at improving quality?

A qualitative study of the Michigan collaborative quality initiatives

Interview topic guide

20.08.2017

Learning for improvement

45. How do you know if you are achieving improvement, and how do you know if that improvement is attributable to your interventions?

46. How do hospitals learn from each other in Michigan? How is learning maximised?

47. How is learning used to improve quality? How does this happen in practice?

48. What are the key features of the Michigan approach that ensure learning is captured and retained?

49. How does the collaborative build organizational memory, and how is it accessed by participants over time?

50. Are there any components of the collaborative you think might be less effective? If you had to drop anything, what would it be, and why?

Academic approach

51. The collaboratives publish a lot of their results as research papers. Was this a planned feature of the collaborative from the outset? Was this a rationale/incentive for setting up the collaborative in the first place? Why?

52. What role does this academic approach play in conferring legitimacy for the collaborative's purpose and activities? Does the importance of this vary between participants, e.g. between program leaders and front-line clinicians? How does it vary?

53. What role do research outputs play in creating and sustaining engagement and motivation among clinicians?

A qualitative study of the Michigan collaborative quality initiatives

Interview topic guide

20.08.2017

Role of competition in improvement

54. Does a sense of competition – for example between hospitals -- have a role in improving quality in Michigan? Why or why not?

55. Do sites or individuals involved in the collaborative feel like they are in competition with each other? Why or why not?

56. At what level is competition most relevant or keenly felt, e.g. between hospitals, between units, between individuals?

Role of collaboration in improvement

57. What role does direct collaboration between participating sites or clinicians play in improvement?

Role of innovation in improvement

58. What is the role of innovation in how hospitals improve? Is innovation an important part of how improvement happens? What is distinctive about innovation in Michigan?

4. Questions to identify features of context

Aim: Identify the program theory of the Michigan CQIs, including identification of specific interventions and their contextual influences, in so doing characterising the mechanisms of their success.

A qualitative study of the Michigan collaborative quality initiatives

Interview topic guide

20.08.2017

General contextual influences

59. What factors do you think make your approach to improvement more or less likely to work?

60. What factors influence the effectiveness of the interventions for improvement?

61. Thinking about the factors which influence success, what do you think are the minimum prerequisites for a collaborative approach to improving quality to work?

Political and policy environment

62. What factors in the political or policy environment in Michigan affect how improvement happens?

63. Have political or policy factors changed over time? How has that impacted improvement?

64. Please tell me about the relationship between the insurer (BCBSM) and the collaborative.

Culture and social environment

65. Please tell me about the culture and ethos in Michigan and in your collaborative in particular. What are they, where do they come from and how are they sustained? What are the cultural hallmarks of the Michigan approach to improvement? To what extent are they shared within the community of participants?

66. How do partners in the collaborative build trust?

A qualitative study of the Michigan collaborative quality initiatives

Interview topic guide

20.08.2017

Leadership

67. What role does leadership play in improvement in Michigan? How important is clinical leadership in particular?

68. How is the collaborative led? How are the culture and ethos of the collaborative reflected in how it is led?

Clinical engagement

69. In the early days, how were clinicians persuaded to engage with a new approach to improvement?

70. Did the methods used to engage clinicians change over time?

71. Are the factors important in achieving and sustaining clinical engagement different? What keeps clinicians engaged and motivated once they are ‘bought in’?

72. How are clinicians persuaded and motivated to change practice, and adopt the practice of other hospitals or clinicians? How is that loss of autonomy managed?

73. Are any hospitals or clinicians disengaged? Why?

Innovation

74. How is a supportive environment for innovation created in Michigan?

75. What features of the environment are important to enable innovation?

A qualitative study of the Michigan collaborative quality initiatives

Interview topic guide

20.08.2017

5. Questions about generalizability

Aim: Generate learning about the distinctive features of the Michigan approach for generalizability and reproduction of improvement in other settings.

76. Would the Michigan approach work anywhere? Why or why not?

77. What advice would you give to other healthcare organizations thinking about adopting a collaborative approach to quality improvement?

78. Would your advice change if that organization was in a different healthcare system, such as the UK?

79. What do think are the most important features of the Michigan approach to improvement, without which it would be impossible to reproduce its success elsewhere?

80. In your experience, what were the most important challenges to improving quality and how were they overcome?

81. What is missing from the Michigan approach that could improve it further? How would you like to see it develop in the future?

6. Safety net

A qualitative study of the Michigan collaborative quality initiatives

Interview topic guide

20.08.2017

82. Is there something else about how and why quality and safety have improved in Michigan you would like to add?
