# Supplementary material for: What are the features of high-performing quality improvement collaboratives? A qualitative case study of a state-wide collaboratives programme
Source: BMJ Open. 2023 Dec 13;13(12):e076648. doi: 10.1136/bmjopen-2023-076648 (PMC10729078; doi:10.1136/bmjopen-2023-076648)
Supplement: Supplementary data [file bmjopen-2023-076648supp001.pdf]

Supplementary Table 1. Summary of distinctive features of the CQIs programme, associated data categories and examples of supporting data

Feature	Data categories	Illustrative supporting data
**Learning from positive deviance**	*Making knowledge accessible to support improvement*	*When you identify the outliers as high performers, you have an opportunity to ask them, what is your technique, for whether it's a procedure technique or a follow-up technique or getting your patients to come back for further data points down the road, you have an opportunity to learn from them (Surgeon)*
	Learning from the collaborative community	*So for example, Flint, Michigan is a town with a lot of challenges, but one of the two big hospitals there provided the best acute care surgery in the state. So that is a reason to celebrate and to have them, [a] high performer, teach the rest of us. (Surgeon)*
**High-quality coordination**	*Offloading administrative and technical burdens from local hospitals and clinicians*	*We do the data analysis for them…So, they'll send in their data and we'll make it pretty for them… So, we just don't shove data to them and say, 'hey, do the best you can, but you've got to improve.' We actually provide face-to-face quality training. We will go into the practice and do mentorship. We'll go into the practice and consult on the practice, if they're really struggling. And then we provide resources for them, so that they don't have to start*

		with white pieces of paper, to initiate their quality improvement project. So, we give them a lot of tools [to] help (Manager)<br><br>*They [the CQI coordinating centre] will assist you in analysing your data from your baseline reporting period, and say you have a higher utilisation, length of stay, ED visit or readmission rate than the rest of the collaborative, [the coordinating centre] would encourage you to focus on these things. (Nurse)*
	Coordinating knowledge exchange	*We are constantly hooking sites up, so today I'm trying to hook, for example, a site up who is doing a certain procedure for Medicare patients. They're having a hard time getting it approved so I went in the database and I looked through to see who else is doing this same procedure for this insurance population, so then I reached out to that coordinator and said "hey, we have a site who's having a hard time. Would you mind sharing anything that's been helpful?" So we do a lot of this. (Nurse)*
**High-quality measurement and comparative**	*Building confidence in data through clinical*	*These collaboratives are developed by the physicians, and..it's their own data, so they believe it. (Manager)*

**performance feedback**	*ownership and standardisation*	*My preferred data to share is how we compare to the other hospitals. And my confidence in doing that is higher with that particular data because whether I'm an abstractor in a Detroit hospital or in a hospital in northern Michigan, we still have to follow the same definitions, abstract the same way. And once the surgeons understood that they did not question the validity of my data as much. (Nurse)*
	Identifying improvement opportunities	*Well, if you don't know how you're performing compared to seventy-two other hospitals, how can you effect change? (Surgeon)*<br><br>*If you have an increase or a high number of SSIs [surgical site infections], then they [the CQI coordinating centre] will recommend that you focus on reducing surgical site infections, and these are some of the ways that you can do it. So not everybody does the same thing, it depends on where they're lacking. (Nurse)*
	Creating accountability for quality	*Two surgeons go [to the service being audited], we send them [the audited service] a formal report back and then they have to stand up at the next collaborative [meeting] within three-to-six months [to] show us their performance improvement plan and where they are. (Surgeon)*

**Careful use of motivational levers**	*Motivating participants to improve*	*What I would say in terms of motivation is there's a lot of motivational levers and that part of running the CQI is that no one lever works for every single person. (Surgeon)*
	Safeguards to preserve healthy competition	*Part of the trust that we've been able to establish, and we have emphasised over and over again that Blue Cross, Blue Shield of Michigan will never see their data. (Manager)*
**Mobilising professional leadership and building community**	*Building relationships and community*	*Fundamentally, they get to see each other and they actually become a community of care givers who know each other and interact, have enough interactions with each other that it really strengthens the coherence of the delivery system. (Manager)*
	Preserving an environment of trust	*They always start their meeting with a general statement, like a confidentiality statement in the presentation, you're all here, we didn't ask you to sign it, but we expect you to adhere to this understanding. (Manager)*
	Preserving professional autonomy	*The fact that they [clinicians] are the ones doing their own interventions to try to make things better, I also consider a success factor of this. They're not told, you've got to use X model to be able to improve this. They are given, I think, complete*

		autonomy as to how they make the improvements in their own area. (Manager)
