# Supplementary material for: What are the features of high-performing quality improvement collaboratives? A qualitative case study of a state-wide collaboratives programme
Source: BMJ Open. 2023 Dec 13;13(12):e076648. doi: 10.1136/bmjopen-2023-076648 (PMC10729078; doi:10.1136/bmjopen-2023-076648)
Supplement: Author's manuscript [file bmjopen-2023-076648.draft_revisions.pdf]

BMJ Open

BMJ Open is committed to open peer review. As part of this commitment we make the peer review history of every article we publish publicly available.

When an article is published we post the peer reviewers' comments and the authors' responses online. We also post the versions of the paper that were used during peer review. These are the versions that the peer review comments apply to.

The versions of the paper that follow are the versions that were submitted during the peer review process. They are not the versions of record or the final published versions. They should not be cited or distributed as the published version of this manuscript.

BMJ Open is an open access journal and the full, final, typeset and author-corrected version of record of the manuscript is available on our site with no access controls, subscription charges or pay-per-view fees (http://bmjopen.bmj.com).

If you have any questions on BMJ Open's open peer review process please email info.bmjopen@bmj.com

BMJ Open

Optimising collaborative quality improvement: qualitative study of a high-performing regional collaboratives programme

Journal:	*BMJ Open*
Manuscript ID	bmjopen-2023-076648
Article Type:	Original research
Date Submitted by the Author:	13-Jun-2023
Complete List of Authors:	McGowan, James; The Healthcare Improvement Studies Institute, Department of Public Health and Primary Care<br>Martin, Graham; University of Cambridge,<br>Krapohl, Greta; University of Michigan Department of Surgery<br>Campbell, Darrell; University of Michigan, School of Medicine<br>Dimick, Justin; University of Michigan, Center for Healthcare Outcomes & Policy, Department of Surgery<br>Dixon-Woods, Mary ; University of Cambridge Primary Care Unit, THIS Institute (The Healthcare Improvement Studies Institute)
Keywords:	Quality in health care < HEALTH SERVICES ADMINISTRATION & MANAGEMENT, HEALTH SERVICES ADMINISTRATION & MANAGEMENT, Organisation of health services < HEALTH SERVICES ADMINISTRATION & MANAGEMENT

SCHOLARONE™
Manuscripts

I, the Submitting Author has the right to grant and does grant on behalf of all authors of the Work (as defined in the below author licence), an exclusive licence and/or a non-exclusive licence for contributions from authors who are: i) UK Crown employees; ii) where BMJ has agreed a CC-BY licence shall apply, and/or iii) in accordance with the terms applicable for US Federal Government officers or employees acting as part of their official duties; on a worldwide, perpetual, irrevocable, royalty-free basis to BMJ Publishing Group Ltd ("BMJ") its licensees and where the relevant Journal is co-owned by BMJ to the co-owners of the Journal, to publish the Work in this journal and any other BMJ products and to exploit all rights, as set out in our licence.

The Submitting Author accepts and understands that any supply made under these terms is made by BMJ to the Submitting Author unless you are acting as an employee on behalf of your employer or a postgraduate student of an affiliated institution which is paying any applicable article publishing charge ("APC") for Open Access articles. Where the Submitting Author wishes to make the Work available on an Open Access basis (and intends to pay the relevant APC), the terms of reuse of such Open Access shall be governed by a Creative Commons licence – details of these licences and which Creative Commons licence will apply to this Work are set out in our licence referred to above.

Other than as permitted in any relevant BMJ Author's Self Archiving Policies, I confirm this Work has not been accepted for publication elsewhere, is not being considered for publication elsewhere and does not duplicate material already published. I confirm all authors consent to publication of this Work and authorise the granting of this licence.

For peer review only

TITLE

Optimising collaborative quality improvement: qualitative study of a high-performing regional collaboratives programme

For peer review only

For peer review only - http://bmjopen.bmj.com/site/about/guidelines.xhtml

ABSTRACT

Objectives: Despite their widespread use, the evidence base for quality improvement collaboratives remains mixed. One reason for this lies in lack of clarity about the drivers of success. We aimed to develop a thematic framework identifying the features of high performance in a regional collaboratives programme that has demonstrated sustained improvements in quality of care in a range of clinical specialties.

Design: Qualitative case study involving interviews with purposively-sampled participants, observations, and analysis of documents.

Setting: The Michigan Collaborative Quality Initiatives (CQIs).

Participants: 38 participants, including clinicians and managers from 10 collaboratives, and staff from University of Michigan and Blue Cross Blue Shield of Michigan.

Results: We identified five features that characterised success in the collaboratives programme: learning from positive deviance; high-quality coordination; high-quality measurement and comparative performance feedback; careful use of motivational levers; and mobilising professional leadership and building community. Rigorous measurement, securing professional leadership and engagement, cultivating a collaborative culture, creating accountability for quality, and relieving participating sites of unnecessary burdens associated with programme participation were all important to high performance.

Conclusions: Our thematic framework offers valuable learning for optimising collaboration-based approaches to improvement in healthcare. These findings have implications for the design, structure and resourcing of quality improvement collaboratives and are likely to be useful to policymakers and health system leaders.

For peer review only

For peer review only - http://bmjopen.bmj.com/site/about/guidelines.xhtml

STRENGTHS AND LIMITATIONS OF THIS STUDY

Strengths

- Identifying the drivers of successful quality improvement collaboratives is a priority for healthcare policymakers, clinicians and researchers
- The use of a qualitative methods with purposive sampling of participants was appropriate to our goal of characterising the features of a high-performing collaboratives programme
- Our sample of clinicians and managers was constructed to capture variability in perspectives among participants

Limitations

- Practical considerations meant it was not possible to sample from all collaboratives in the programme; purposive sampling of participants meant that some selection bias may have occurred;
- Our study did not include patient perspectives, which may have differed from those of the study participants

For peer review only

For peer review only - http://bmjopen.bmj.com/site/about/guidelines.xhtml

INTRODUCTION

Often addressing areas of clinical practice with known variation in care processes or outcomes, quality improvement collaboratives seek to bring together healthcare professionals from multiple settings to address shared improvement goals.[1 2] Though the structure of collaboratives can vary, they typically involve participating clinicians and organisations receiving performance feedback, usually benchmarked against data from other sites,[3 4] and attending meetings to share learning and identify interventions that might be used to support improvement. Despite their widespread use as a mechanism to accelerate the uptake of practice innovations to improve care,[5] the evidence base for the effectiveness of collaboration-based approaches to improvement is mixed.[1-4 6-9]

One challenge in improving the consistency with which collaboratives deliver on their goals arises from ongoing uncertainty regarding the underlying drivers of performance of collaboratives, and lack of clarity around "what good looks like".[10-14] An emerging literature[15-17] suggests that understanding what characterises high performance is likely to help in addressing this problem. The literature, including research on high reliability organisations,[18 19] the "Safety II" movement (which emphasises the need to investigate how things can and do go well)[20-23] and the body of work on the importance of recognising and understanding "positive deviance,"[24] is united by its emphasis on the value of producing learning by identifying the features of high performance and making them accessible and replicable. An example is the use of positive deviance approaches [24 25] to address unwarranted variation in processes of care, as in the well-known example of door-to-balloon studies for cardiac catheterisation.[26 27]

In this article, we report a qualitative case study based on interviews, observation and documentary analysis that aimed to identify the distinctive features of a regional collaboratives

For peer review only - http://bmjopen.bmj.com/site/about/guidelines.xhtml

programme that has demonstrated success over a long period, outperforming secular trends to improve care in a variety of clinical specialties and domains of quality.[28-32]

METHODS

We conducted a qualitative case study of a regional collaboratives programme: the Michigan Collaborative Quality Initiatives (CQIs).[33]

Case study

Funded by Blue Cross Blue Shield of Michigan, the state-wide CQIs programme currently operates 18 quality improvement collaboratives across a range of medical and surgical specialties. Participation among providers in the state is high.[34] Focusing on care processes associated with high volume, cost and variation in practice, the programme is an important example of a collaborative model that has achieved sustained improvements in quality and safety.[28] Our case study was based on qualitative analysis of interview, observational and programme document data. The study was deemed exempt from review by the Health Sciences and Behavioral Sciences Institutional Review Board at University of Michigan (IRB-HSBS).

Participants and sampling

We purposively sampled clinicians and managers across a spectrum from more mature to less well-established collaboratives. We sought to capture variation across state geography, socioeconomic status of patient population served, hospital size, urban and rural settings, and academic and non-academic hospitals. We included participants from the host organisation of the collaboratives' coordinating centers (University of Michigan) and from the programme funder (Blue Cross Blue Shield of Michigan).

For peer review only - http://bmjopen.bmj.com/site/about/guidelines.xhtml

Data collection

Interviews were conducted in person or remotely (by telephone) at coordinating center sites and participating hospitals in Michigan. We obtained written informed consent from all participants. Interviews were digitally recorded using an encrypted voice recorder and were supported by a topic guide that was informed by literature review and discussion within the project team. Audio recordings were transcribed and transcripts were anonymised. Participant observation was conducted by JM during a quality improvement conference hosted by one of the CQIs, supported by contemporaneous digital recordings which were subsequently transcribed, anonymised and included in the analysis. Programme documents were collected and treated as data.

Data analysis

Data management was supported by NVivo software (version 10). Data analysis was based on the constant comparative method.[35] Each interview transcript was read, coded and re-coded multiple times to describe each relevant unit of meaning. A thematic framework was developed that was iteratively refined, theorised and applied to other transcripts and documents to synthesise findings.

Ethics

The study was deemed exempt from review by the Health Sciences and Behavioral Sciences Institutional Review Board at University of Michigan (IRB-HSBS).

Patient and public involvement

This was an interview-based qualitative study of a regional quality improvement collaboratives program, involving clinicians and managers who participated in the collaboratives. Patients and the public were not involved in this study.

For peer review only

For peer review only - http://bmjopen.bmj.com/site/about/guidelines.xhtml

RESULTS

We included 38 interview participants (clinicians and managers from 10 CQIs, University of Michigan and Blue Cross Blue Shield of Michigan: **Table 1**), 12 hours of participant observation and 12 programme documents in our study.

Table 1. Descriptive characteristics of study participants.

Characteristics	Number of participants
By primary professional role	
Physicians	15
Nurses	11
Managers	12
By organisation	
Michigan Surgical Quality Collaborative	15
Michigan Bariatric Surgery Collaborative	5
Blue Cross Blue Shield of Michigan	5
Michigan Arthroplasty Registry Collaborative Quality Initiative	2
Michigan Urological Surgery Improvement Collaborative	2
Michigan Oncology Quality Consortium	2

For peer review only - http://bmjopen.bmj.com/site/about/guidelines.xhtml

University of Michigan Quality Department	2
Michigan Trauma Quality Improvement Programme	1
Michigan Radiation Oncology Quality Consortium	1
Michigan Anticoagulation Quality Improvement Initiative	1
Michigan Society of Thoracic and Cardiovascular Surgeons Quality Collaborative	1
Michigan Cardiovascular Consortium	1
**Total**	**38**

Source: Authors' analysis of case study data

Our analysis identified five distinctive features implicated in the success of the CQIs programme: learning from positive deviance; high-quality coordination; high-quality measurement and comparative performance feedback; careful use of motivational levers; and mobilising professional leadership and building community. **Table 2** summarises the features and themes identified with supporting data.

Table 2. Summary of distinctive features of the CQIs programme, associated themes and supporting data

Feature	Theme	Supporting data

For peer review only - http://bmjopen.bmj.com/site/about/guidelines.xhtml

**Learning from positive deviance**	*Making knowledge accessible to support improvement*	*So when you identify the outliers as high performers, you have an opportunity to ask them, what is your technique, for whether it's a procedure technique or a follow-up technique or getting your patients to come back for further data points down the road, you have an opportunity to learn from them …(T18)*
	Learning from the collaborative community	*So for example, Flint, Michigan is a town with a lot of challenges, but one of the two big hospitals there provided the best acute care surgery in the state. So it's like, oh, that is a reason to celebrate and to have them, high performer, teach the rest of us.(T8)*
**High-quality coordination**	*Offloading administrative and technical burdens from local sites*	*We do the data analysis for them…So, they'll send in their data and we'll make it pretty for them… So, we just don't shove data to them and say, 'hey, do the best you can, but you've got to improve.' We actually provide face to face quality training. We will go into the practice and do mentorship. We'll go into the practice and consult on the practice, if they're really struggling. And then we provide resources for them, so that they don't have to start with white pieces of paper, to kind of initiate their quality improvement project. So, we give them a lot of tools help both with engagement, but success. (T20)*

For peer review only - http://bmjopen.bmj.com/site/about/guidelines.xhtml

	Coordinating knowledge exchange	*And then we are constantly hooking sites up, so today I'm trying to hook, for example, a site up who is doing a certain procedure for Medicare patients. They're having a hard time getting it approved so I went in the database and I looked through to see, okay, who else is doing this same procedure for this insurance population, okay, so then I reached out to that coordinator and said, hey, we have a site who's having a hard time. Would you mind sharing anything that's been helpful? So we do a lot of this.(T17)*
**High-quality measurement and comparative performance feedback**	*Building confidence in data through clinical ownership*	*These collaboratives are developed by the physicians, and by far it's their own data, so they believe it. (T31)*
	Identifying improvement opportunities	*Well, if you don't know how you're performing compared to seventy-two other hospitals, how can you effect change? (T5)*
	Creating accountability for quality	*..two surgeons go, we send them a formal report back and then they have to stand up at the next collaborative or the next one, so within three-to-six months show us their performance improvement plan and where they are.(T23)*

For peer review only - http://bmjopen.bmj.com/site/about/guidelines.xhtml

**Careful use of motivational levers**	*Motivating participants to improve*	*What I would say in terms of motivation is there's a lot of motivational levers and that part of running the CQI is that no one lever works for every single person. (T1)*
	Safeguards to preserve healthy competition	*..part of the trust that we've been able to establish, and we have emphasised over and over again that Blue Cross, Blue Shield of Michigan will never see their data. (T19)*
**Mobilising professional leadership and building community**	*Building relationships and community*	*Really important because fundamentally, they get to see each other and they actually become a community of care givers who know each other and interact, have enough interactions with each other that it really strengthens, essentially, the coherence of the delivery system.(T10)*
	Preserving an environment of trust	*They always start their meeting with kind of a general statement, like a confidentiality kind of like statement in the presentation, like, you're all here, we didn't ask you to sign it, but we expect you to kind of adhere to this understanding.(T12)*
	Preserving professional autonomy	*The fact that they are the ones doing their own interventions to try to make things better, I also consider a success factor of this. They're not told, you've got to use X model to be able to improve this. They are given, I think, complete autonomy*

For peer review only - http://bmjopen.bmj.com/site/about/guidelines.xhtml

		as to how they make the improvements in their own area.(T29)

Source: Authors' analysis of case study data

Learning from positive deviance

The collaboratives did not seek to promote a particular quality improvement methodology. Instead, consistent with the principles of positive deviance, [25] they focused on sourcing effective interventions to address specific quality problems from within the community, and then making them easy to share and implement widely. Accordingly, participants described the collaborative 'theory of change' as identifying positive outliers on measures of quality (which may be organisations, teams, or individual physicians), learning from them, making accessible the knowledge and practices associated with high performance, and then promoting standardisation of practices to improve quality across the collaborative as a whole.

We do it more, positive deviancy stories, and that's pretty common, I think, to most of the collaboratives. Try and go for people who have done, who are doing something really well, who have really exemplary performance...on outcomes […] We systematise some of the knowledge, and present it back. (T2)

In seeking out the clinical and management practices associated with high-quality care, collaborative leaders aimed to demonstrate the value of mutual learning, role-modelled openness to learning from colleagues and sites across the state, and deliberately enfranchised participants from non-academic centers and those not traditionally perceived as high-performing.

There are folks out there who do a phenomenal hip, there are folks out there who do a phenomenal colon. They don't have to be in a university set-up…Then, let's go to these places

For peer review only - http://bmjopen.bmj.com/site/about/guidelines.xhtml

where we see an outlier surgeon doing exceptionally well, and see what he's doing different. (T3)

High-quality coordination

The organisation and actions of coordinating centers were consistently identified as important to the success of the collaboratives. Comprising clinically-led teams with expertise in quality improvement, these centers met the definition of coordination as "integrating or linking together different parts of an organisation to accomplish a collective set of tasks."[36] One of their important roles was that of resolving mundane challenges that might otherwise undermine participation in collaborative quality improvement, for example by providing technical support to data abstractors and clinicians at hospital sites, addressing data collection problems, conducting analysis and interpretation of clinical registry data, and supporting evaluation of interventions. By absorbing much of the hard work involved in improvement, including administrative tasks, coordinating centers reduced the load on staff at local sites.

We're trying to spoon feed it as much as possible…so that the administrative burden is lifted from the site. Meaning if there is a specific QI intervention that requires, I don't know, printing something and making something and distributing, that we try to offload that as much as possible. (T33)

Participants proposed that by centralising responsibility for labor-intensive or technically challenging tasks such as data analysis, coordinating centers enabled staff at participating sites to focus on innovation and implementation of improvement interventions. The centers also functioned to coordinate networks of knowledge-sharing and exchange, linking sites with shared quality challenges together with the aim of accelerating the sharing of practices. They therefore played a critical role in marshalling the knowledge offered by local sites, sustaining commitment, and coordinating cooperation among participating organisations and clinicians.

For peer review only - http://bmjopen.bmj.com/site/about/guidelines.xhtml

We hook them up with somebody who was an outlier in the past who's not an outlier anymore, say, you might want to talk to so-and-so, because often the problem is not not knowing the data, it's how do you apply best practices in your patient workflow. (T24)

High-quality measurement and comparative performance feedback

Participants emphasised that high-quality measurement and data was a prerequisite for successful collaborative quality improvement. Without credible data, participants reported that clinicians could neither reliably identify quality challenges, nor track the extent to which interventions (including those codified by positive deviance learning) had an impact. To secure legitimacy among clinicians, the collaboratives engaged deeply in processes to foster clinical ownership of data collection and analysis processes, underpinned by rigorous approaches to standardisation, risk-adjustment, audit and quality assurance. Registry data then provided clinicians with a 'warrant for action' to change practice locally.

I have total confidence that I can go to my hospital or my people and say, this is real, we have a wound infection rate of 5.6, that's real, believe me, it's risk-adjusted, you can trust it, we got to work at it, there's a problem. (T30)

Comparative performance feedback was key to making data meaningful and in creating accountability for quality among participating organisations and clinicians. Alongside the provision of data on relative performance such as provider and site-level rankings, forums for delivering feedback provided opportunities for participating clinicians to hold one another accountable for quality. These forums—including collaborative meetings, site visits and independent peer review of clinical practices—were also used to identify improvement opportunities and evaluate quality at organisation and individual physician level.

For peer review only - http://bmjopen.bmj.com/site/about/guidelines.xhtml

The leader of the coordinating center will stand up and go, okay, Dr X, we see that you don't have…a very good rate on your SSI [surgical site infections], so what's going on there? I mean, call them out. (T6)

Careful use of motivational levers

The collaboratives employed a range of motivational levers to drive behavior change among clinicians, including competitiveness, peer pressure, and professional esteem. Healthy competition was seen as a key driver of improvement as clinicians were keen to be recognised by their peers as performing well.

There's nothing like a little bit of healthy competition. When a general surgeon sees that they're the lowest among, you know, their counterparts, that stimulates some serious, you know, ownership of that, and motivation to get better. (T16)

Besides their efforts to motivate participants, the collaboratives safeguarded friendly competition by introducing protections against anti-collaborative behaviors, thereby moderating the potential dark sides of excessive competitive tendencies. This was achieved, for example, by setting terms of reference that made clear that clinical registry data could not be used to gain commercial competitive advantage outside the collaborative community.

The other thing was that people wouldn't use the data for competitive advantage, so that you would know your data, but you wouldn't be able to know who the other people were, and that nobody in the collaborative was allowed to publish or present the data or use the data at all from the collaborative to say that they did something better than some other group. (T26)

Mobilising professional leadership and building community

For peer review only - http://bmjopen.bmj.com/site/about/guidelines.xhtml

The collaboratives provided a formal framework through which clinicians could rally around a common purpose, giving expression to an underlying motivation to improve care for patients as a community of professionals. This required the creation and maintenance of an environment of trust, achieved by setting clear boundaries regarding data use, insulating improvement activities from the influence of insurance companies, building on pre-existing social networks, and protecting clinicians' autonomy.

They're the ones who see the data and the data does not go back to the insurance company. It's not used to decide where patients are directed. It's used to help surgeons to enable them to improve, and so that by really making sure that people began to trust the process and kind of following through on that and engaging in it they were making the decisions about what we were doing. (T4)

The collaboratives cultivated high levels of trust between participating clinicians, giving them license to experiment and to deviate from established practices in pursuit of ambitious improvement goals. Being part of a collaborative community provided protections necessary to take calculated risks to improve patient care.

[In reference to an innovative practice] Sites bought into that because they had trust to say, if everyone else in the group is doing this, I'm willing to go along as well. Even though I'm worried it may cause problems with bleeding, and this and that, I'm willing to give it a try because I trust the rest of this group enough. (T9)

DISCUSSION

Our study has identified five features of a high-performing regional collaboratives programme that may be implicated in its success: learning from positive deviance; high-quality coordination; high-quality measurement and comparative performance feedback; careful use of motivational levers; and mobilising professional leadership and building community. The

For peer review only - http://bmjopen.bmj.com/site/about/guidelines.xhtml

findings are likely to be useful to policymakers and healthcare systems in understanding how collaboration-based approaches can be optimised to maximise impact on quality of care,[2 4] may help to address the persistent evidence gap about the features of collaboratives that drive performance,[9 11] and could enable better replication and scaling of successful models.[37-39]

Our study underscores the importance of high-quality measurement in persuading clinicians of the veracity and actionability of quality problems. Long a problem for those using administrative data sources for improvement work,[40-42] a key challenge lies in ensuring that data are perceived by those being asked to act on it as valid, reliable and clinically relevant.[43] Doubt about the accuracy of data can lead to skepticism about the reality of problems and about the value of proposed solutions, or to loss of confidence in the improvement programme.[43-46] Accordingly, those leading collaboratives should focus on maximising the credibility of data used for improvement work. This might be achieved by ensuring data collection is aligned with the goals of participating professionals, is based on clinical rather than administrative data sources, and by bestowing responsibility for data quality upon clinicians. Building on existing evidence of the advantages of securing professional leadership for data collection and the development of improvement interventions,[43 47] such an approach may support collaborative improvement efforts by strengthening clinical ownership.[48 49]

Consistent with previous research on the challenges of measurement for clinical teams,[50] our study indicates that the task of securing clinical engagement for collaborative quality improvement is best achieved when the many mundane but highly consequential practical and technical challenges of data collection and analysis are taken care of in a competent way that relieves burden from participating clinicians.[47 50] By providing the knowledge and technical skills needed to collect, analyse and interpret clinical data for use in quality improvement,[51] collaboratives can address the persistent problem of shortages of local analytical capability,[50 52] support the identification of practices associated with high performance, and gain the

For peer review only - http://bmjopen.bmj.com/site/about/guidelines.xhtml

confidence of clinicians. Analytics and improvement knowledge of this kind is increasingly recognised as an important form of expertise in its own right, and one which specialists other than clinicians may be best placed to provide.[53-55]

Equally important to successful collaboratives are conditions that enable a 'collaborative culture', including professional commitment to improvement and a spirit of collegial competitiveness, to flourish.[4 5 10 56 57] A key lesson from our analysis is that making these levers work well meant taking active steps to ensure that more malign forms of competitiveness were excluded.[58] For example, safeguards such as a ban on use of registry data to gain commercial advantage and on sharing unblinded performance data with insurance companies helped to preserve the collaboratives as communities where professional collegiality was the guiding principle. By identifying the role of protections against behaviors that undermine collaboration, our study highlights how collaboratives might successfully neutralise anti-competitive elements of programmes that can undermine improvement[58 59] while activating the power of professional communities by insulating them from the negative effects of markets and bureaucracies.[60 61]

Finally, structures that create accountability for quality among clinicians appears to be critical.[5 56 62-64] In our case study, forums that brought participating clinicians together created 'obligations to explain and justify conduct',[65 66] establishing social relations that facilitated accountability for improving quality. This feature of the collaboratives was consistent with theory from social science that 'accountability requires a forum'[65]—a setting in which to *perform* accountability through social interaction—and may be especially valuable in enhancing the effectiveness of performance feedback as an improvement strategy.

Our study has limitations. First, the use of qualitative methods and purposive sampling of participants, though appropriate to our goal of characterising the features of the collaboratives, necessitated a small sample of stakeholders, meaning that some selection bias may have occurred. Second, although our sample was intended to capture variability in perspectives

For peer review only - http://bmjopen.bmj.com/site/about/guidelines.xhtml

among participants, practical considerations meant it was not possible to sample from all CQIs in the programme. Finally, our study did not include patient perspectives, which may have differed from those of participants; eliciting them may represent both a locus for improving the value of the CQIs programme and a focus of future research.

CONCLUSIONS

Our study has identified "what good looks like" for high-performing quality improvement collaboratives, including distinctive features of a regional programme. Our findings have implications for the design, structure and resourcing of quality improvement collaboratives both in the US and internationally, and may inform efforts to replicate and scale successful models. Of particular importance are the need for rigorous measurement, securing professional leadership and engagement, cultivating a collaborative culture, creating accountability for quality, and ensuring that collaborative infrastructures are adequately resourced to relieve participating sites of the burdens of improvement work. Just as important as technical and administrative support is the building of the right kind of community ethos: high-quality collaboration relies on norms and rules of engagement that enable both cooperation and healthy competition to flourish.

For peer review only

COMPETING INTERESTS

The Michigan Quality Collaboratives are supported by Blue Cross and Blue Shield of Michigan. MJE receives salary support from Blue Cross and Blue Shield of Michigan as well as the NIH and the Michigan DHHS. DCC, GK and MJE's institution (University of Michigan) receives a partial salary paid for by the Blue Cross Blue Shield of Michigan Value Partnerships for the Michigan Surgical Quality Collaborative. GK, DCC and MJE are employed by the Michigan Surgical Quality Collaborative. MJE and JBD were interviewed as participants in the study.

FUNDING

This study was funded by James McGowan's NIHR Academic Clinical Fellowship, by Mary Dixon-Woods' Wellcome Trust Investigator award (WT097899) and by the Health Foundation's grant to the University of Cambridge for The Healthcare Improvement Studies (THIS) Institute. THIS Institute is supported by the Health Foundation—an independent charity committed to bringing about better health and health care for people in the UK. MDW is a National Institute for Health Research (NIHR) Senior Investigator (NF-SI-0617-10026).

The funders had no role in the design and conduct of the study; collection, management, analysis, and interpretation of the data; preparation, review, or approval of the manuscript; and decision to submit the manuscript for publication.

The views expressed in this article are those of the authors and not necessarily those of the NHS, the NIHR, or the Department of Health and Social Care.

AUTHORS' CONTRIBUTIONS

JM had full access to all of the data in the study and takes responsibility for the integrity of the data and the accuracy of the data analysis.

For peer review only - http://bmjopen.bmj.com/site/about/guidelines.xhtml

Concept and design: JM, GK, DCC, MDW

Acquisition, analysis, or interpretation of data: JM, GM, GK, MDW

Drafting of the manuscript: JM, GM, MDW

Critical revision of the manuscript for important intellectual content: All authors

Statistical analysis: Not applicable

Obtained funding: JM, MDW

Administrative, technical, or material support: JM, GK, DCC, MJE, MDW

Supervision: MDW

For peer review only

For peer review only - http://bmjopen.bmj.com/site/about/guidelines.xhtml

REFERENCES

1. De Silva D. Improvement collaboratives in health care. London: The Health Foundation 2014.
2. Hulscher M, Schouten L, Grol R. Collaboratives. London: The Health Foundation. Quest for Quality and Improved Performance 2009.
3. Schouten LM, Hulscher ME, van Everdingen JJ, Huijsman R, Grol RP. Evidence for the impact of quality improvement collaboratives: systematic review. BMJ 2008;**336**(7659):1491-94.
4. Øvretveit J, Bate P, Cleary P, et al. Quality collaboratives: lessons from research. Qual Saf Health Care 2002;**11**(4):345-51.
5. Nix M, McNamara P, Genevro J, et al. Learning Collaboratives: Insights And A New Taxonomy From AHRQ's Two Decades Of Experience. Health Affairs 2018;**37**(2):205-12 doi: 10.1377/hlthaff.2017.1144.
6. Wells S, Tamir O, Gray J, Naidoo D, Bekhit M, Goldmann D. Are quality improvement collaboratives effective? A systematic review. BMJ Qual Saf 2018;**27**(3):226-40.
7. Power M, Tyrrell PJ, Rudd AG, et al. Did a quality improvement collaborative make stroke care better? A cluster randomized trial. Implementation Science 2014;**9**(1):40 doi: 10.1186/1748-5908-9-40.
8. Schouten LMT, Grol RPTM, Hulscher MEJL. Factors influencing success in quality-improvement collaboratives: development and psychometric testing of an instrument. Implementation Science 2010;**5**(1):84 doi: 10.1186/1748-5908-5-84.
9. Martin G, Dixon-Woods M. Collaboration-Based Approaches. Cambridge: Cambridge University Press, 2022.
10. Leroy L, Rittner JL, Johnson KE, Gerteis J, Miller T. Facilitative Components of Collaborative Learning: A Review of Nine Health Research Networks. Healthc Policy 2017;**12**(3):19-33.
11. Nadeem E, Olin SS, Hill LC, Hoagwood KE, Horwitz SM. Understanding the components of quality improvement collaboratives: a systematic literature review. The Milbank Quarterly 2013;**91**(2):354-94.
12. Hulscher MEJL, Schouten LMT, Grol RPTM, Buchan H. Determinants of success of quality improvement collaboratives: what does the literature show? BMJ Quality & Safety 2013;**22**(1):19-31 doi: 10.1136/bmjqs-2011-000651.
13. Ranmuthugala G, Plumb JJ, Cunningham FC, Georgiou A, Westbrook JI, Braithwaite J. How and why are communities of practice established in the healthcare sector? A systematic review of the literature. BMC Health Services Research 2011;**11**(1):273 doi: 10.1186/1472-6963-11-273.
14. Zamboni K, Baker U, Tyagi M, Schellenberg J, Hill Z, Hanson C. How and under what circumstances do quality improvement collaboratives lead to better outcomes? A systematic review. Implementation Science 2020;**15**(1):27 doi: 10.1186/s13012-020-0978-z.
15. Lawton R, Taylor N, Clay-Williams R, Braithwaite J. Positive deviance: a different approach to achieving patient safety. BMJ Qual Saf 2014;**23**(11):880-83.
16. Liberati EG, Tarrant C, Willars J, et al. Seven features of safety in maternity units: a framework based on multisite ethnography and stakeholder consultation. BMJ Quality & Safety 2020:bmjqs-2020-010988 doi: 10.1136/bmjqs-2020-010988.
17. Bradley EH, Curry LA, Ramanadhan S, Rowe L, Nembhard IM, Krumholz HM. Research in action: using positive deviance to improve quality of health care. Implementation science 2009;**4**(1):25.
18. Weick KE, Sutcliffe KM, Obstfeld D. Organizing for high reliability: Processes of collective mindfulness. Crisis management 1999;**3**:81-123.

For peer review only - http://bmjopen.bmj.com/site/about/guidelines.xhtml

19. Sutcliffe KM, Paine L, Pronovost PJ. Re-examining high reliability: actively organising for safety. BMJ Quality & Safety 2017;**26**(3):248-51 doi: 10.1136/bmjqs-2015-004698.
20. Le Coze JC. Vive la diversité! High Reliability Organisation (HRO) and Resilience Engineering (RE). Safety Science 2019;**117**:469-78 doi: https://doi.org/10.1016/j.ssci.2016.04.006.
21. Hollnagel E. *Safety-II in Practice: Developing the Resilience Potentials*. 1st ed: Routledge, 2017.
22. Hollnagel E, Braithwaite J, Wears RL. *Delivering Resilient Health Care*: Routledge, 2018.
23. Braithwaite J, Wears RL, Hollnagel E. Resilient health care: turning patient safety on its head. International Journal for Quality in Health Care 2015;**27**(5):418-20 doi: 10.1093/intqhc/mzv063.
24. Baxter R, Taylor N, Kellar I, Lawton R. What methods are used to apply positive deviance within healthcare organisations? A systematic review. BMJ Qual Saf 2016;**25**(3):190-201 doi: 10.1136/bmjqs-2015-004386 [published Online First: 2015/11/22].
25. O'Hara JK, Grasic K, Gutacker N, et al. Identifying positive deviants in healthcare quality and safety: a mixed methods study. Journal of the Royal Society of Medicine 2018;**111**(8):276-91 doi: 10.1177/0141076818772230.
26. Bradley EH, Curry LA, Ramanadhan S, Rowe L, Nembhard IM, Krumholz HM. Research in action: using positive deviance to improve quality of health care. Implement Sci 2009;**4**:25 doi: 10.1186/1748-5908-4-25 [published Online First: 2009/05/12].
27. Bradley EH, Curry LA, Webster TR, et al. Achieving Rapid Door-To-Balloon Times: How Top Hospitals Improve Complex Clinical Systems. Circulation 2006;**113**(8):1079-85 doi: 10.1161/CIRCULATIONAHA.105.590133.
28. Share DA, Campbell DA, Birkmeyer N, et al. How a regional collaborative of hospitals and physicians in Michigan cut costs and improved the quality of care. Health Affairs 2011;**30**(4):636-45.
29. Hurley P, Dhir A, Gao Y, et al. A Statewide Intervention Improves Appropriate Imaging in Localized Prostate Cancer. J Urol 2017;**197**(5):1222-28 doi: 10.1016/j.juro.2016.11.098 [published Online First: 20161123].
30. Womble PR, Linsell SM, Gao Y, et al. A statewide intervention to reduce hospitalizations after prostate biopsy. The Journal of Urology 2015;**194**(2):403-09.
31. Milojevic M, Bond C, Theurer PF, et al. The Role of Regional Collaboratives in Quality Improvement: Time to Organize, and How? Seminars in Thoracic and Cardiovascular Surgery 2020;**32**(1):8-13 doi: https://doi.org/10.1053/j.semtcvs.2019.07.013.
32. Vu JV, Collins SD, Seese E, et al. Evidence that a Regional Surgical Collaborative Can Transform Care: Surgical Site Infection Prevention Practices for Colectomy in Michigan. Journal of the American College of Surgeons 2018;**226**(1):91-99 doi: 10.1016/j.jamcollsurg.2017.10.013.
33. Collaborative Quality Initiatives. Secondary Collaborative Quality Initiatives 2021. https://www.valuepartnerships.com/programs/collaborative-quality-initiatives/.
34. Member Hospitals. Secondary Member Hospitals 2021. https://msqc.org/about/member-hospitals/.
35. Charmaz K. *Constructing grounded theory: A practical guide through qualitative analysis*: Sage, 2006.
36. Van De Ven AH, Delbecq AL, Koenig R. Determinants of Coordination Modes within Organizations. American Sociological Review 1976;**41**(2):322-38 doi: 10.2307/2094477.
37. Howarth E, Devers K, Moore G, O'Cathain A, Dixon-Woods M. Contextual issues and qualitative research. Challenges, solutions and future directions in the evaluation of service innovations in health care and public health: NIHR Journals Library, 2016.
38. Craig P, Dieppe P, Macintyre S, Michie S, Nazareth I, Petticrew M. Developing and evaluating complex interventions: the new Medical Research Council guidance. Bmj 2008;**337**:a1655.

For peer review only - http://bmjopen.bmj.com/site/about/guidelines.xhtml

39. Sheetz KH, Englesbe MJ. Expanding the Quality Collaborative Model as a Blueprint for Higher-Value Care. JAMA Health Forum 2020;**1**(5):e200413-e13 doi: 10.1001/jamahealthforum.2020.0413.
40. Ghaferi AA, Dimick JB. Practical Guide to Surgical Data Sets: Medicare Claims Data. JAMA Surgery 2018;**153**(7):677-78 doi: 10.1001/jamasurg.2018.0489.
41. Lawson EH, Louie R, Zingmond DS, et al. A Comparison of Clinical Registry Versus Administrative Claims Data for Reporting of 30-Day Surgical Complications. Annals of Surgery 2012;**256**(6):973-81 doi: 10.1097/SLA.0b013e31826b4c4f.
42. Osborne NH, Nicholas LH, Ryan AM, Thumma JR, Dimick JB. Association of hospital participation in a quality reporting program with surgical outcomes and expenditures for Medicare beneficiaries. Jama 2015;**313**(5):496-504.
43. Dixon-Woods M, McNicol S, Martin G. Ten challenges in improving quality in healthcare: lessons from the Health Foundation's programme evaluations and relevant literature. BMJ Quality & Safety 2012;**21**(10):876-84.
44. Ling T. *How do you get clinicians involved in quality improvement?: an evaluation of the Health Foundation's engaging with quality initiative: a programme of work to support clinicians to drive forward quality*: The Health Foundation, 2010.
45. Benning A, Ghaleb M, Suokas A, et al. Large scale organisational intervention to improve patient safety in four UK hospitals: mixed method evaluation. Bmj 2011;**342**:d195.
46. Benn J, Burnett S, Parand A, Pinto A, Iskander S, Vincent C. The journey to safety: a report of 24 NHS organisations undertaking the Safer Patients Initiative. 2011.
47. Dixon-Woods M, Campbell A, Aveling E-L, Martin G. An ethnographic study of improving data collection and completeness in large-scale data exercises. Wellcome Open Res 2019;**4**:203-03 doi: 10.12688/wellcomeopenres.14993.1.
48. Mountford J, Shojania KG. Refocusing quality measurement to best support quality improvement: local ownership of quality measurement by clinicians. BMJ Quality & Safety 2012;**21**(6):519-23 doi: 10.1136/bmjqs-2012-000859.
49. Campbell DA. Quality Improvement Is Local. Journal of the American College of Surgeons 2009;**209**(1):141-43 doi: https://doi.org/10.1016/j.jamcollsurg.2009.03.012.
50. Woodcock T, Liberati EG, Dixon-Woods M. A mixed-methods study of challenges experienced by clinical teams in measuring improvement. BMJ Quality & Safety 2021;**30**(2):106-15 doi: 10.1136/bmjqs-2018-009048.
51. Health Foundation. Learning report: Safer Patients Initiative. Lessons from the first major improvement programme addressing patient safety in the UK. 2011.
52. Bardsley M. Understanding analytical capability in health care. Do we have more data than insight? London: Health Foundation, 2016.
53. Lillo-Crespo M, Sierras-Davó MC, Taylor A, Ritters K, Karapostoli A. Mapping the Status of Healthcare Improvement Science through a Narrative Review in Six European Countries. International Journal of Environmental Research and Public Health 2019;**16**(22):4480.
54. Wright D, Gabbay J, Le May A. Determining the skills needed by frontline NHS staff to deliver quality improvement: findings from six case studies. BMJ Quality & Safety 2022;**31**(6):450-61 doi: 10.1136/bmjqs-2021-013065.
55. Martin GP. Beyond improvement skills: what do clinicians, managers, patients and others need to do to make improvement happen? BMJ Quality & Safety 2022;**31**(6):423-25 doi: 10.1136/bmjqs-2021-014181.
56. Young GJ. Multistakeholder Regional Collaboratives Have Been Key Drivers Of Public Reporting, But Now Face Challenges. Health Affairs 2012;**31**(3):578-84.
57. Krapohl GL, Hemmila MR, Hendren S, et al. Building, scaling, and sustaining a learning health system for surgical quality improvement: A toolkit. Learning Health Systems 2020;**4**(3):e10215 doi: https://doi.org/10.1002/lrh2.10215.
58. Carter P, Ozieranski P, McNicol S, Power M, Dixon-Woods M. How collaborative are quality improvement collaboratives: a qualitative study in stroke care. Implementation Science 2014;**9**(1):32 doi: 10.1186/1748-5908-9-32.

For peer review only - http://bmjopen.bmj.com/site/about/guidelines.xhtml

59. Freidson E. *Professionalism, the third logic: On the practice of knowledge*: University of Chicago press, 2001.
60. Martin GP, Armstrong N, Aveling E-L, Herbert G, Dixon-Woods M. Professionalism Redundant, Reshaped, or Reinvigorated? Realizing the "Third Logic" in Contemporary Health Care. Journal of Health and Social Behavior 2015;**56**(3):378-97 doi: 10.1177/0022146515596353.
61. Evetts J. New Professionalism and New Public Management: Changes, Continuities and Consequences. Comparative Sociology 2009;**8**(2):247-66 doi: https://doi.org/10.1163/156913309X421655.
62. Dückers MLA, Wagner C, Vos L, Groenewegen PP. Understanding organisational development, sustainability, and diffusion of innovations within hospitals participating in a multilevel quality collaborative. Implementation Science 2011;**6**(1):18 doi: 10.1186/1748-5908-6-18.
63. Parand A, Benn J, Burnett S, Pinto A, Vincent C. Strategies for sustaining a quality improvement collaborative and its patient safety gains. International Journal for Quality in Health Care 2012;**24**(4):380-90 doi: 10.1093/intqhc/mzs030.
64. Dixon-Woods M, Bosk CL, Aveling E-L, Goeschel C, Pronovost PJ. Explaining Michigan: Developing an Ex Post Theory of a Quality Improvement Program. The Milbank Quarterly 2011;**89**(2):167-205 doi: https://doi.org/10.1111/j.1468-0009.2011.00625.x.
65. Bovens M. Analysing and assessing accountability: A conceptual framework. European Law Journal 2007;**13**(4):447-68.
66. Bovens M, Schillemans T, Hart Pt. Does public accountability work? An assessment tool. Public Administration 2008;**86**(1):225-42.

For peer review only

For peer review only - http://bmjopen.bmj.com/site/about/guidelines.xhtml

Standards for Reporting Qualitative Research (SRQR)

Manuscript title: Optimising collaborative quality improvement: qualitative study of a high-performing regional collaboratives programme

Title and abstract

	Addressed	Page(s).
**Title** - Concise description of the nature and topic of the study Identifying the study as qualitative or indicating the approach (e.g., ethnography, grounded theory) or data collection methods (e.g., interview, focus group) is recommended	Yes	1
**Abstract** - Summary of key elements of the study using the abstract format of the intended publication; typically includes background, purpose, methods, results, and conclusions	Yes	2

Introduction

**Problem formulation** - Description and significance of the problem/phenomenon studied; review of relevant theory and empirical work; problem statement	Yes	4-5
**Purpose or research question** - Purpose of the study and specific objectives or questions	Yes	4-5

Methods

**Qualitative approach and research paradigm** - Qualitative approach (e.g., ethnography, grounded theory, case study, phenomenology, narrative research) and guiding theory if appropriate; identifying the research paradigm (e.g., postpositivist, constructivist/ interpretivist) is also recommended; rationale**	Yes	4-6
**Researcher characteristics and reflexivity** - Researchers' characteristics that may influence the research, including personal attributes, qualifications/experience, relationship with participants, assumptions, and/or presuppositions; potential or actual interaction between researchers' characteristics and the research questions, approach, methods, results, and/or transferability	No	See cover letter
**Context** - Setting/site and salient contextual factors; rationale**	Yes	5-6
**Sampling strategy** - How and why research participants, documents, or events were selected; criteria for deciding when no further sampling was necessary (e.g., sampling saturation); rationale**	Yes	5-6
**Ethical issues pertaining to human subjects** - Documentation of approval by an appropriate ethics review board and participant consent, or explanation for lack thereof; other confidentiality and data security issues	Yes	5-6
**Data collection methods** - Types of data collected; details of data collection procedures including (as appropriate) start and stop dates of data collection and analysis, iterative process, triangulation of sources/methods, and modification of procedures in response to evolving study findings; rationale**	Yes	5-6

For peer review only

For peer review only - http://bmjopen.bmj.com/site/about/guidelines.xhtml

**Data collection instruments and technologies** - Description of instruments (e.g., interview guides, questionnaires) and devices (e.g., audio recorders) used for data collection; if/how the instrument(s) changed over the course of the study	Yes	5-6
**Units of study** - Number and relevant characteristics of participants, documents, or events included in the study; level of participation (could be reported in results)	Yes	5-8
**Data processing** - Methods for processing data prior to and during analysis, including transcription, data entry, data management and security, verification of data integrity, data coding, and anonymization/de-identification of excerpts	Yes	5-6
**Data analysis** - Process by which inferences, themes, etc., were identified and developed, including the researchers involved in data analysis; usually references a specific paradigm or approach; rationale**	Yes	5-6
**Techniques to enhance trustworthiness** - Techniques to enhance trustworthiness and credibility of data analysis (e.g., member checking, audit trail, triangulation); rationale**	Yes	5-6

Results/findings

**Synthesis and interpretation** - Main findings (e.g., interpretations, inferences, and themes); might include development of a theory or model, or integration with prior research or theory	Yes	7-16
**Links to empirical data** - Evidence (e.g., quotes, field notes, text excerpts, photographs) to substantiate analytic findings	Yes	7-16

Discussion

**Integration with prior work, implications, transferability, and contribution(s) to the field** - Short summary of main findings; explanation of how findings and conclusions connect to, support, elaborate on, or challenge conclusions of earlier scholarship; discussion of scope of application/generalizability; identification of unique contribution(s) to scholarship in a discipline or field	Yes	16-19
**Limitations** - Trustworthiness and limitations of findings	Yes	3 & 16-19

Other

**Conflicts of interest** - Potential sources of influence or perceived influence on study conduct and conclusions; how these were managed	Yes	20
**Funding** - Sources of funding and other support; role of funders in data collection, interpretation, and reporting	Yes	20

For peer review only

For peer review only - http://bmjopen.bmj.com/site/about/guidelines.xhtml

What are the features of high-performing quality improvement collaboratives? A qualitative case study of a state-wide collaboratives programme

Journal:	*BMJ Open*
Manuscript ID	bmjopen-2023-076648.R1
Article Type:	Original research
Date Submitted by the Author:	16-Oct-2023
Complete List of Authors:	McGowan, James; University of Cambridge, THIS Institute (The Healthcare Improvement Studies Institute)<br>Martin, Graham; University of Cambridge, THIS Institute (The Healthcare Improvement Studies Institute)<br>Krapohl, Greta; University of Michigan, Department of Surgery<br>Campbell, Darrell; University of Michigan, Department of Surgery<br>Englesbe, M; University of Michigan, Department of Surgery<br>Dimick, Justin; University of Michigan, Department of Surgery<br>Dixon-Woods, Mary ; University of Cambridge, THIS Institute (The Healthcare Improvement Studies Institute)
**Primary Subject Heading**:	Health policy
Secondary Subject Heading:	Health policy, Health services research, Medical management, Public health, Qualitative research
Keywords:	Quality in health care < HEALTH SERVICES ADMINISTRATION & MANAGEMENT, HEALTH SERVICES ADMINISTRATION & MANAGEMENT, Organisation of health services < HEALTH SERVICES ADMINISTRATION & MANAGEMENT, Health policy < HEALTH SERVICES ADMINISTRATION & MANAGEMENT, Public Hospitals < Hospitals, Public

I, the Submitting Author has the right to grant and does grant on behalf of all authors of the Work (as defined in the below author licence), an exclusive licence and/or a non-exclusive licence for contributions from authors who are: i) UK Crown employees; ii) where BMJ has agreed a CC-BY licence shall apply, and/or iii) in accordance with the terms applicable for US Federal Government officers or employees acting as part of their official duties; on a worldwide, perpetual, irrevocable, royalty-free basis to BMJ Publishing Group Ltd ("BMJ") its licensees and where the relevant Journal is co-owned by BMJ to the co-owners of the Journal, to publish the Work in this journal and any other BMJ products and to exploit all rights, as set out in our licence.

The Submitting Author accepts and understands that any supply made under these terms is made by BMJ to the Submitting Author unless you are acting as an employee on behalf of your employer or a postgraduate student of an affiliated institution which is paying any applicable article publishing charge ("APC") for Open Access articles. Where the Submitting Author wishes to make the Work available on an Open Access basis (and intends to pay the relevant APC), the terms of reuse of such Open Access shall be governed by a Creative Commons licence – details of these licences and which Creative Commons licence will apply to this Work are set out in our licence referred to above.

Other than as permitted in any relevant BMJ Author's Self Archiving Policies, I confirm this Work has not been accepted for publication elsewhere, is not being considered for publication elsewhere and does not duplicate material already published. I confirm all authors consent to publication of this Work and authorise the granting of this licence.

For peer review only

BMJ Open_v2.0_2023-10-13

What are the features of high-performing quality improvement collaboratives? A qualitative case study of a state-wide collaboratives programme

ABSTRACT

Objectives: Despite their widespread use, the evidence base for the effectiveness of quality improvement collaboratives remains mixed. Lack of clarity about "what good looks like" in collaboratives remains a persistent problem. We aimed to identify the distinctive features of a state-wide collaboratives programme that has demonstrated sustained improvements in quality of care in a range of clinical specialties over a long period.

Design: Qualitative case study involving interviews with purposively sampled participants, observations, and analysis of documents.

Setting: The Michigan Collaborative Quality Initiatives (CQIs) programme.

Participants: 38 participants, including clinicians and managers from ten collaboratives, and staff from University of Michigan and Blue Cross Blue Shield of Michigan.

Results: We identified five features that characterised success in the collaboratives programme: learning from positive deviance; high-quality coordination; high-quality measurement and comparative performance feedback; careful use of motivational levers; and mobilising professional leadership and building community. Rigorous measurement, securing professional leadership and engagement, cultivating a collaborative culture, creating accountability for quality, and relieving participating sites of unnecessary burdens associated with programme participation were all important to high performance.

Conclusions: Our findings offer valuable learning for optimising collaboration-based approaches to improvement in healthcare, with implications for the design, structure and resourcing of quality improvement collaboratives. These findings are likely to be useful to

For peer review only

For peer review only - http://bmjopen.bmj.com/site/about/guidelines.xhtml

26 clinicians, managers, policymakers and health system leaders engaged in multi-organisational

27 approaches to improving quality and safety.

For peer review only

For peer review only - http://bmjopen.bmj.com/site/about/guidelines.xhtml

STRENGTHS AND LIMITATIONS OF THIS STUDY

- Identifying the drivers of successful quality improvement collaboratives is a priority for healthcare policymakers, clinicians and researchers.
- A qualitative design was appropriate to our goal of characterising the features of a high-performing collaboratives programme.
- Our sample of clinicians and managers was constructed to capture variability in perspectives among participants.
- Practical considerations meant it was not possible to sample from all collaboratives in the programme; purposive sampling of participants meant that some selection bias may have occurred.
- Our study did not include patient perspectives, which may have differed from those of the study participants.

For peer review only - http://bmjopen.bmj.com/site/about/guidelines.xhtml

INTRODUCTION

Often addressing areas of clinical practice with known variation in care processes or outcomes, or gaps between current and best practice, quality improvement collaboratives seek to bring together healthcare professionals from multiple organisations to address shared improvement goals.[1-4] Though their structure and methods can vary, collaboratives typically seek to provide a mechanism to accelerate the uptake of practice innovations to improve care across multiple clinical settings.[4] Key activities include the provision of performance feedback to participating clinicians and organisations usually benchmarked against data from other sites,[5-7] and face-to-face meetings to share learning and identify interventions that might be used to support and scale improvement.[2] Building on early models such as the Northern New England Cardiovascular Disease Study Group and Vermont Oxford Network in the 1980s,[5, 8, 9] and more recent approaches such as the Institute for Healthcare Improvement's Breakthrough Series design,[10] collaboration-based approaches have emerged as a prominent strategy for improving healthcare quality and safety across OECD countries, including in the United States.[3, 11] Despite their widespread use across a variety of clinical specialties and organisational contexts, the evidence base for the effectiveness of improvement collaboratives is mixed.[1-3, 5-7, 12, 13]

One challenge in improving the consistency with which collaboratives improve quality of care arises from ongoing uncertainty regarding the underlying drivers of their performance, including lack of clarity about "what good looks like".[11, 14-17] An emerging literature [18-20] suggests that understanding what characterises high performance is likely to help in addressing this problem. The literature, including research on high reliability organisations,[21, 22] the "Safety II" movement (which emphasises the need to investigate how things can and do go well)[23-26] and the body of work on the importance of recognising and understanding

For peer review only - http://bmjopen.bmj.com/site/about/guidelines.xhtml

"positive deviance,"[27] is united by its emphasis on the value of producing learning by identifying the features of high performance and making them accessible and replicable. An example is the use of positive deviance approaches [27, 28] to address unwarranted variation in processes of care, as in the well-known example of door-to-balloon studies for cardiac catheterisation.[20, 29]

In this article, we report a qualitative case study of a regional (state-wide) collaboratives programme that has demonstrated high performance over a long period: the Michigan Collaborative Quality Initiatives (CQIs) programme.[30] Funded by Blue Cross Blue Shield of Michigan, the programme operates multiple quality improvement collaboratives across a range of medical and surgical specialties. Focusing on care processes associated with high volume, cost and variation in practice, the programme is an important example of a collaborative model that has achieved sustained improvements in quality and safety, outperforming secular trends and improvements made by other improvement programmes.[31-35] Between 2005 and 2009, for example, hospitals participating in the general surgery collaborative reduced risk-adjusted morbidity by 2.6%, outperforming results achieved by the American College of Surgeons' National Surgical Quality Improvement Program (NSQIP).[31] Similarly, hospitals participating in the bariatric collaborative reduced risk-adjusted mortality faster than hospitals outside Michigan that participated in NSQIP during the same period.[31] More recent improvements have been reported by the urological [32, 33] and cardiothoracic surgery [34] collaboratives.

We aimed to identify distinctive features of this state-wide programme that might be implicated in success and thereby build understanding of what good looks like for quality improvement collaboratives.

METHODS

For peer review only

We conducted a qualitative case study of the CQIs programme based on semi-structured interviews with participating clinicians and managers, observation of a quality improvement meeting of the Michigan Surgical Quality Collaborative (the largest and longest-standing CQI), and analysis of programme documents.

Sampling and recruitment

We purposively sampled clinicians and managers across a spectrum from more mature to less well-established collaboratives. We sought to capture variation across a range of participant characteristics, including state geography, socioeconomic status of patient population served, size of participating hospital, urban and rural settings, and academic and non-academic hospitals. As a very high proportion of collaboratives in the CQI programme are surgical in nature, the ability to sample clinical participants from non-surgical specialties was more limited.

We also sampled participants from the host organisation of the collaboratives' coordinating centres (University of Michigan) and from the programme funder (Blue Cross Blue Shield of Michigan). A letter of invitation and study information document were sent to potential interview participants identified by GLK, all of whom were offered an opportunity to discuss the study with JM before consenting to be interviewed.

For peer review only

Data collection

Interviews were conducted in person or remotely (by telephone) by one interviewer (JM), who was not known to participants, at coordinating centre sites and participating hospitals. We obtained written informed consent from all participants. Interviews were digitally recorded using an encrypted voice recorder and were supported by a topic guide (**supplemental file**) that was informed by literature review and discussion within the project team. Audio recordings were transcribed and transcripts were anonymised.

For peer review only - http://bmjopen.bmj.com/site/about/guidelines.xhtml

114 Additionally, participant observation was conducted by JM during a Michigan Surgical Quality Collaborative (MSQC) meeting. JM attended individual sessions, observed presentations and interactions, and talked informally with other attendees about the work of the collaborative. This was supported by contemporaneous digital recordings made by JM which were subsequently transcribed, anonymised and included in the analysis. Programme documents were collected during the MSQC meeting and treated as data.

Analysis

Data management was supported by NVivo software (version 10). Data analysis was based on the constant comparative method.[36] Each interview transcript was read, coded and re-coded multiple times to describe each relevant unit of meaning. Comparisons were made between data, codes and categories in order to identify distinct analytic concepts in the data and relationships between them. A thematic framework was developed that was iteratively refined, theorised and applied to the data to synthesise findings. Data analysis and interpretation and development of the thematic framework was led by JM with support from GM and MDW.

Ethics

The study was deemed exempt from review by the Health Sciences and Behavioral Sciences Institutional Review Board at University of Michigan (IRB-HSBS).

Patient and public involvement

None.

For peer review only

RESULTS

We included 38 interview participants (clinicians and managers from ten CQIs, University of Michigan and Blue Cross Blue Shield of Michigan; **Table 1**), 12 hours of participant observation and 12 programme documents in our study.

Table 1. Descriptive characteristics of interview participants

Characteristic	Number of participants
Professional role	
Surgeons and Physicians	15
Nurses	11
Programme managers	12
Organisation	
Michigan Surgical Quality Collaborative	15
Michigan Bariatric Surgery Collaborative	5
Blue Cross Blue Shield of Michigan	5
Michigan Arthroplasty Registry Collaborative Quality Initiative	2
Michigan Urological Surgery Improvement Collaborative	2
Michigan Oncology Quality Consortium	2

For peer review only

For peer review only - http://bmjopen.bmj.com/site/about/guidelines.xhtml

University of Michigan Quality Department	2
Michigan Trauma Quality Improvement Programme	1
Michigan Radiation Oncology Quality Consortium	1
Michigan Anticoagulation Quality Improvement Initiative	1
Michigan Society of Thoracic and Cardiovascular Surgeons Quality Collaborative	1
Michigan Cardiovascular Consortium	1
**Total**	**38**

Our analysis identified five distinctive features implicated in the success of the CQIs programme: learning from positive deviance; high-quality coordination; high-quality measurement and comparative performance feedback; careful use of motivational levers; and mobilising professional leadership and building community. **Supplementary Table 1** summarises the features and data categories identified by our analysis with supporting data.

Learning from positive deviance

The collaboratives did not seek to promote a particular quality improvement methodology. Instead, consistent with the principles of positive deviance,[28] they focused on sourcing effective interventions to address specific quality problems from within the community, and

For peer review only - http://bmjopen.bmj.com/site/about/guidelines.xhtml

then making them easy to share and implement widely. Accordingly, participants described the collaborative 'theory of change' as identifying positive outliers on measures of quality (which may be organisations, whole clinical teams, or individual care providers), learning from them, making accessible the knowledge and practices associated with their high performance, and then promoting standardisation of practices to improve quality across the collaborative as a whole.

We do it more, positive deviancy stories, and that's pretty common, I think, to most of the collaboratives. Try and go for people who have done, who are doing something really well, who have really exemplary performance...on outcomes […] We systematise some of the knowledge, and present it back. (Surgeon)

In seeking out the clinical and management practices associated with high-quality care, collaborative leaders aimed to demonstrate the value of mutual learning, role-modelled openness to learning from colleagues and sites across the state, and deliberately enfranchised participants from non-academic centres and those not traditionally perceived as high-performing.

There are folks out there who do a phenomenal hip, there are folks out there who do a phenomenal colon. They don't have to be in a university set-up…Then, let's go to these places where we see an outlier surgeon doing exceptionally well, and see what he's doing different. (Surgeon)

High-quality coordination

The organisation and actions of coordinating centres were consistently identified as important to the success of the collaboratives. Comprising clinically-led teams with expertise in quality improvement, these centres met the definition of coordination as "integrating or linking together different parts of an organisation to accomplish a collective set of tasks."[37] One of their important roles was that of resolving mundane challenges that might otherwise

For peer review only - http://bmjopen.bmj.com/site/about/guidelines.xhtml

undermine participation in collaborative quality improvement, for example by providing technical support to data abstractors and clinicians at hospital sites, addressing data collection problems, conducting analysis and interpretation of clinical registry data, and supporting evaluation of interventions. By absorbing much of the hard work involved in improvement, including administrative tasks, coordinating centres reduced the load on staff at local sites.

When they [the coordinating centre] are asking the abstractors to collect the information out of the patients' charts to put it into the MSQC [Michigan Surgical Quality Collaborative] workstation, they have done tremendous hard work in making sure that they do not present it to the abstractors until it is truly ready to go in a useable form and that if we have questions, they have already done the hard work of answering those questions. (Nurse)

Participants proposed that by centralising responsibility for labour-intensive or technically challenging tasks such as data analysis, coordinating centres enabled staff at participating sites to focus on innovation and implementation of improvement interventions. The centres also functioned to coordinate networks of knowledge-sharing and exchange, linking sites with shared quality challenges together with the aim of accelerating the sharing of practices. They therefore played a critical role in marshalling the knowledge offered by local sites, sustaining commitment, and coordinating cooperation among participating organisations and clinicians.

We hook them up with somebody who was an outlier in the past who's not an outlier anymore, say, you might want to talk to so-and-so, because often the problem is not not knowing the data, it's how do you apply best practices in your patient workflow. (Physician)

High-quality measurement and comparative performance feedback

Participants emphasised that high-quality measurement and data was a prerequisite for successful collaborative quality improvement. Without credible data, participants reported that

For peer review only - http://bmjopen.bmj.com/site/about/guidelines.xhtml

199 clinicians could neither reliably identify quality challenges, nor track the extent to which
200 interventions (including those codified by positive deviance learning) had an impact. To secure
201 legitimacy among clinicians, the collaboratives engaged deeply in processes to foster clinical
202 ownership of data collection and analysis processes, underpinned by rigorous approaches to
203 standardisation, risk-adjustment, audit and quality assurance. Registry data then provided
204 clinicians with a ‘warrant for action’ to change practice locally.

205 *I have total confidence that I can go to my hospital or my people and say, this is real, we have*
206 *a wound infection rate of 5.6, that’s real, believe me, it’s risk-adjusted, you can trust it, we got*
207 *to work at it, there’s a problem. (Surgeon)*

208 Comparative performance feedback was key to making data meaningful and in creating
209 accountability for quality among clinical teams in participating hospitals. Alongside the
210 provision of data on relative performance such as comparisons between providers and
211 hospital-level rankings, forums for delivering feedback provided opportunities for participating
212 clinicians to hold one another accountable for quality. These forums—including collaborative
213 meetings, site visits, and independent peer review of clinical practices—were also used to
214 identify improvement opportunities and evaluate quality at hospital and care provider level.

215 *The leader of the coordinating centre will stand up and go, okay, Dr X, we see that you don’t*
216 *have a very good rate on your SSI [surgical site infections], so what’s going on there? I mean,*
217 *call them out. (Nurse)*

218 Careful use of motivational levers

219 The collaboratives employed a range of motivational levers to drive behaviour change among
220 clinicians, including competitiveness, peer pressure, and professional esteem. Healthy
221 competition was seen as a key driver of improvement as clinicians were keen to be recognised
222 by their peers as performing well.

For peer review only - http://bmjopen.bmj.com/site/about/guidelines.xhtml

There's nothing like a little bit of healthy competition. When a general surgeon sees that they're the lowest among their counterparts, that stimulates some serious ownership of that, and motivation to get better. (Nurse)

Besides their efforts to motivate participants, the collaboratives safeguarded friendly competition by introducing protections against anti-collaborative behaviours, thereby moderating the potential dark sides of excessive competitive tendencies. This was achieved, for example, by setting terms of reference that made clear that clinical registry data could not be used to gain commercial competitive advantage outside the collaborative community.

The other thing was that people wouldn't use the data for competitive advantage, so that you would know your data, but you wouldn't be able to know who the other people were, and that nobody in the collaborative was allowed to publish or present the data or use the data at all from the collaborative to say that they did something better than some other group. (Surgeon)

Mobilising professional leadership and building community

The collaboratives provided a formal framework through which clinicians could rally around a common purpose, giving expression to an underlying motivation to improve care for patients as a community of professionals. This required the creation and maintenance of an environment of trust, achieved by setting clear boundaries regarding data use, insulating improvement activities from the influence of insurance companies, building on pre-existing social networks, and protecting clinicians' autonomy.

They're the ones who see the data and the data does not go back to the insurance company. It's not used to decide where patients are directed. It's used to help surgeons to enable them to improve, and so that by really making sure that people began to trust the process and kind

For peer review only

of following through on that and engaging in it they were making the decisions about what we were doing. (Surgeon)

The collaboratives cultivated high levels of trust between participating clinicians, giving them licence to experiment and to deviate from established practices in pursuit of ambitious improvement goals. Being part of a collaborative community provided protections necessary to take calculated risks to improve patient care.

[In reference to an innovative practice] Sites bought into that because they had trust to say, "if everyone else in the group is doing this, I'm willing to go along as well. Even though I'm worried it may cause problems with bleeding, and this and that, I'm willing to give it a try because I trust the rest of this group enough." (Physician)

DISCUSSION

Our study has identified five features of a high-performing state-wide collaboratives programme that may be implicated in its success: learning from positive deviance; high-quality coordination; high-quality measurement and comparative performance feedback; careful use of motivational levers; and mobilising professional leadership and building community. By providing insight into what good looks like, the findings are likely to be useful to policymakers and healthcare systems in understanding how collaboration-based approaches can be optimised to maximise impact on quality of care, may help to address the persistent evidence gap about the features of collaboratives that drive performance, and could enable better replication and scaling of successful models.[38-40]

Our study underscores the importance of high-quality measurement in persuading clinicians of the veracity and actionability of quality problems. Broadly understood as the means by which performance in relation to quality of care can be reliably ascertained,[41, 42] high-quality

For peer review only - http://bmjopen.bmj.com/site/about/guidelines.xhtml

For peer review only

measurement has long been recognised as foundational to successful quality improvement efforts.[43-45] A well-established challenge for those using administrative data sources for improvement work[46-48] lies in ensuring that data are perceived by those being asked to act on it as valid, reliable and clinically relevant.[42] Doubts about data can lead to scepticism about the reality of problems and about the value of proposed solutions, or to loss of confidence in the improvement programme.[49-52] By implementing rigorous, clinically-led processes to risk-adjust and quality assure the data used for improvement work, the Michigan collaboratives succeeded in avoiding many of the problems commonly associated with quality measurement [42, 53] including the potentially weak risk-adjustment often characteristic of administrative or insurance-based data sources,[48] problems with case capture and data completeness, [43, 44, 54], and variation in data collection systems.[41, 42, 55]

Accordingly, our findings indicate that those leading collaboratives should focus on optimising the credibility of data used for improvement. This might be achieved by ensuring data collection is aligned with the goals of participating professionals, and by bestowing responsibility for data quality upon clinicians. Building on existing evidence of the advantages of securing professional leadership for data collection and the development of improvement interventions,[56] such an approach might better support collaborative improvement by strengthening clinical ownership.[54, 57]

Consistent with previous research on the challenges of measurement for clinical teams, our study indicates that the task of securing clinical engagement for collaborative quality improvement is best achieved when the many mundane but highly consequential practical and technical challenges of data collection and analysis are taken care of in a competent way that relieves burden from participating clinicians.[43, 54, 56] By providing the knowledge and technical skills needed to collect, analyse and interpret clinical data for use in quality improvement,[58] collaboratives can address the persistent problem of shortages of local analytical capability,[43, 59] support the identification of practices associated with high performance, and gain the confidence of clinicians. Analytics and improvement knowledge of

For peer review only - http://bmjopen.bmj.com/site/about/guidelines.xhtml

297 this kind is increasingly recognised as an important form of expertise in its own right, and one
298 that specialists other than clinicians may be best placed to provide.[60-62]

299 Equally important to successful collaboratives are conditions that enable a 'collaborative
300 culture', including professional commitment to improvement and a spirit of collegial
301 competitiveness, to flourish.[4, 6, 14, 63, 64] Previous research has drawn attention to the
302 limitations of audit and performance feedback as an improvement strategy in healthcare,[65]
303 including leveraging competition between hospitals and providers as stimulus for
304 improvement.[66-69] A key lesson from our analysis is that making these levers work well
305 meant taking active steps to ensure that more malign forms of competitiveness were excluded.
306 For example, safeguards such as a ban on use of registry data to gain commercial advantage
307 and on sharing unblinded performance data with insurance companies helped to preserve the
308 collaboratives as communities where professional collegiality was the guiding principle. By
309 identifying the role of protections against behaviours that undermine collaboration, our study
310 highlights how collaboratives might successfully neutralise anti-competitive elements of
311 programmes that can undermine improvement,[70, 71] while activating the power of
312 professional communities by insulating them from the negative effects of markets and
313 bureaucracies.[72, 73]

314 Finally, structures that create accountability for quality among clinicians appear to be critical.[4,
315 63, 74-76] In our case study, forums that brought participating clinicians together created
316 'obligations to explain and justify conduct',[77, 78] establishing social relations that facilitated
317 accountability for improving quality. This feature of the collaboratives was consistent with
318 theory from social science that indicates that 'accountability requires a forum'[77]—a setting in
319 which to *perform* accountability through social interaction—and may be especially valuable in
320 enhancing the effectiveness of performance feedback as an improvement strategy.

321 Our study has limitations. First, the use of qualitative methods and purposive sampling of
322 participants, though appropriate to our goal of characterising the features of the collaboratives

For peer review only - http://bmjopen.bmj.com/site/about/guidelines.xhtml

programme, necessitated a small sample of stakeholders, meaning that some selection bias may have occurred. Second, although our sample was intended to capture variability in perspectives among participants, practical considerations meant it was not possible to sample from all CQIs in the programme. Relatedly, surgical collaboratives—particularly the Michigan Surgical Quality Collaborative—and surgeon participants were over-represented in our interview sample, though the sample overall was broadly reflective of the target population (participants in the Michigan CQIs programme), where surgical CQIs accounted for the largest proportion of active collaboratives. Third, in common with all qualitative research, it is possible researcher-related factors biased our analysis in some non-transparent way. Finally, our study did not include patient perspectives, which might have differed from those of participants; eliciting them may represent both a locus for improving the value of the CQIs programme and a focus of future research.

CONCLUSIONS

Our study has identified "what good looks like" for high-performing quality improvement collaboratives, including distinctive features of a state-wide programme. Our findings have implications for the design, structure and resourcing of quality improvement collaboratives both in the US and internationally, and may inform efforts to replicate and scale successful models. Of particular importance are the need for rigorous measurement, securing professional leadership and engagement, cultivating a collaborative culture, creating accountability for quality, and ensuring that collaborative infrastructures are adequately resourced to relieve participating sites of the burdens of improvement work. Just as important as technical and administrative support is the building of the right kind of community ethos: high-quality multi-organisational approaches to improving quality rely on norms and rules of engagement that enable both cooperation and healthy (non-financial) competition to flourish.

For peer review only - http://bmjopen.bmj.com/site/about/guidelines.xhtml

COMPETING INTERESTS

The Michigan Quality Collaboratives are supported by Blue Cross and Blue Shield of Michigan. MJE receives salary support from Blue Cross and Blue Shield of Michigan as well as the NIH and the Michigan DHHS. DCC, GK and MJE's institution (University of Michigan) receives a partial salary paid for by the Blue Cross Blue Shield of Michigan Value Partnerships for the Michigan Surgical Quality Collaborative. GK, DCC and MJE are employed by the Michigan Surgical Quality Collaborative. MJE and JBD were interviewed as participants in the study.

FUNDING

This study was funded by James McGowan's NIHR Academic Clinical Fellowship (ACF-2016-14-011), by Mary Dixon-Woods' Wellcome Trust Investigator award (WT097899) and by the Health Foundation's grant to the University of Cambridge for The Healthcare Improvement Studies (THIS) Institute. THIS Institute is supported by the Health Foundation—an independent charity committed to bringing about better health and health care for people in the UK. MDW is a National Institute for Health Research (NIHR) Senior Investigator (NF-SI-0617-10026).

The funders had no role in the design and conduct of the study; collection, management, analysis, and interpretation of the data; preparation, review, or approval of the manuscript; and decision to submit the manuscript for publication.

The views expressed in this article are those of the authors and not necessarily those of the NHS, the NIHR, or the Department of Health and Social Care.

CONTRIBUTORS

For peer review only - http://bmjopen.bmj.com/site/about/guidelines.xhtml

For peer review only

JM had full access to all of the data in the study and takes responsibility for the integrity of the data and the accuracy of the data analysis. *Concept and design:* JM, GK, DCC, MDW. *Acquisition, analysis, or interpretation of data:* JM, GM, GK, MDW. *Drafting of the manuscript:* JM, GM, MDW. *Critical revision of the manuscript for important intellectual content:* All authors. *Statistical analysis:* Not applicable. *Obtained funding:* JM, MDW. *Administrative, technical, or material support:* JM, GK, DCC, MJE, MDW. *Supervision:* MDW.

DATA AVAILABILITY STATEMENT

No additional data available.

For peer review only

REFERENCES

1. De Silva, D., *Improvement collaboratives in health care.* London: The Health Foundation, 2014.
2. Hulscher, M., L. Schouten, and R. Grol, *Collaboratives. London: The Health Foundation.* Quest for Quality and Improved Performance, 2009.
3. Martin, G. and M. Dixon-Woods, *Collaboration-Based Approaches*. 2022, Cambridge University Press: Cambridge.
4. Nix, M., P. McNamara, J. Genevro, et al., *Learning Collaboratives: Insights And A New Taxonomy From AHRQ's Two Decades Of Experience.* Health Affairs, 2018. **37**(2): p. 205-212.
5. Schouten, L.M., M.E. Hulscher, J.J. van Everdingen, et al., *Evidence for the impact of quality improvement collaboratives: systematic review.* BMJ, 2008. **336**(7659): p. 1491-1494.
6. Øvretveit, J., P. Bate, P. Cleary, et al., *Quality collaboratives: lessons from research.* Qual Saf Health Care, 2002. **11**(4): p. 345-351.
7. Power, M., P.J. Tyrrell, A.G. Rudd, et al., *Did a quality improvement collaborative make stroke care better? A cluster randomized trial.* Implementation Science, 2014. **9**(1): p. 40.
8. Malenka, D.J. and G.T. O'Connor, *The Northern New England Cardiovascular Disease Study Group: A Regional Collaborative Effort for Continuous Quality Improvement in Cardiovascular Disease.* The Joint Commission Journal on Quality Improvement, 1998. **24**(10): p. 594-600.
9. Horbar, J.D., J. Rogowski, P.E. Plsek, et al., *Collaborative Quality Improvement for Neonatal Intensive Care.* Pediatrics, 2001. **107**(1): p. 14-22.
10. Institute for Healthcare Improvement, *The Breakthrough Series: IHI's Collaborative Model for Achieving Breakthrough Improvement.*, in *IHI Innovation Series white paper*. 2003: Boston, MA.
11. Nadeem, E., S.S. Olin, L.C. Hill, et al., *Understanding the components of quality improvement collaboratives: a systematic literature review.* The Milbank Quarterly, 2013. **91**(2): p. 354-394.
12. Wells, S., O. Tamir, J. Gray, et al., *Are quality improvement collaboratives effective? A systematic review.* BMJ Qual Saf, 2018. **27**(3): p. 226-240.
13. Schouten, L.M.T., R.P.T.M. Grol, and M.E.J.L. Hulscher, *Factors influencing success in quality-improvement collaboratives: development and psychometric testing of an instrument.* Implementation Science, 2010. **5**(1): p. 84.
14. Leroy, L., J.L. Rittner, K.E. Johnson, et al., *Facilitative Components of Collaborative Learning: A Review of Nine Health Research Networks.* Healthcare policy = Politiques de sante, 2017. **12**(3): p. 19-33.
15. Hulscher, M.E.J.L., L.M.T. Schouten, R.P.T.M. Grol, et al., *Determinants of success of quality improvement collaboratives: what does the literature show?* BMJ Quality & Safety, 2013. **22**(1): p. 19-31.
16. Ranmuthugala, G., J.J. Plumb, F.C. Cunningham, et al., *How and why are communities of practice established in the healthcare sector? A systematic review of the literature.* BMC Health Services Research, 2011. **11**(1): p. 273.
17. Zamboni, K., U. Baker, M. Tyagi, et al., *How and under what circumstances do quality improvement collaboratives lead to better outcomes? A systematic review.* Implementation Science, 2020. **15**(1): p. 27.

For peer review only - http://bmjopen.bmj.com/site/about/guidelines.xhtml

18. Lawton, R., N. Taylor, R. Clay-Williams, et al., *Positive deviance: a different approach to achieving patient safety.* BMJ Qual Saf, 2014. **23**(11): p. 880-883.
19. Liberati, E.G., C. Tarrant, J. Willars, et al., *Seven features of safety in maternity units: a framework based on multisite ethnography and stakeholder consultation.* BMJ Quality & Safety, 2020: p. bmjqs-2020-010988.
20. Bradley, E.H., L.A. Curry, S. Ramanadhan, et al., *Research in action: using positive deviance to improve quality of health care.* Implementation science, 2009. **4**(1): p. 25.
21. Weick, K.E., K.M. Sutcliffe, and D. Obstfeld, *Organizing for high reliability: Processes of collective mindfulness.* Crisis management, 1999. **3**: p. 81-123.
22. Sutcliffe, K.M., L. Paine, and P.J. Pronovost, *Re-examining high reliability: actively organising for safety.* BMJ Quality & Safety, 2017. **26**(3): p. 248-251.
23. Le Coze, J.C., *Vive la diversité! High Reliability Organisation (HRO) and Resilience Engineering (RE).* Safety Science, 2019. **117**: p. 469-478.
24. Hollnagel, E., *Safety-II in Practice: Developing the Resilience Potentials*. 1st ed. 2017: Routledge.
25. Hollnagel, E., J. Braithwaite, and R.L. Wears, *Delivering Resilient Health Care*. 2018: Routledge.
26. Braithwaite, J., R.L. Wears, and E. Hollnagel, *Resilient health care: turning patient safety on its head.* International Journal for Quality in Health Care, 2015. **27**(5): p. 418-420.
27. Baxter, R., N. Taylor, I. Kellar, et al., *What methods are used to apply positive deviance within healthcare organisations? A systematic review.* BMJ Qual Saf, 2016. **25**(3): p. 190-201.
28. O'Hara, J.K., K. Grasic, N. Gutacker, et al., *Identifying positive deviants in healthcare quality and safety: a mixed methods study.* Journal of the Royal Society of Medicine, 2018. **111**(8): p. 276-291.
29. Bradley, E.H., L.A. Curry, T.R. Webster, et al., *Achieving Rapid Door-To-Balloon Times: How Top Hospitals Improve Complex Clinical Systems.* Circulation, 2006. **113**(8): p. 1079-1085.
30. Blue Cross Blue Shield of Michigan. *Collaborative Quality Initiatives*. 2023 [cited 2023 October 2023]; Available from: https://www.valuepartnerships.com/programs/collaborative-quality-initiatives/.
31. Share, D.A., D.A. Campbell, N. Birkmeyer, et al., *How a regional collaborative of hospitals and physicians in Michigan cut costs and improved the quality of care.* Health Affairs, 2011. **30**(4): p. 636-645.
32. Hurley, P., A. Dhir, Y. Gao, et al., *A Statewide Intervention Improves Appropriate Imaging in Localized Prostate Cancer.* J Urol, 2017. **197**(5): p. 1222-1228.
33. Womble, P.R., S.M. Linsell, Y. Gao, et al., *A statewide intervention to reduce hospitalizations after prostate biopsy.* The Journal of Urology, 2015. **194**(2): p. 403-409.
34. Milojevic, M., C. Bond, P.F. Theurer, et al., *The Role of Regional Collaboratives in Quality Improvement: Time to Organize, and How?* Seminars in Thoracic and Cardiovascular Surgery, 2020. **32**(1): p. 8-13.
35. Vu, J.V., S.D. Collins, E. Seese, et al., *Evidence that a Regional Surgical Collaborative Can Transform Care: Surgical Site Infection Prevention Practices for Colectomy in Michigan.* Journal of the American College of Surgeons, 2018. **226**(1): p. 91-99.
36. Charmaz, K., *Constructing grounded theory: A practical guide through qualitative analysis*. 2006: Sage.
37. Van De Ven, A.H., A.L. Delbecq, and R. Koenig, *Determinants of Coordination Modes within Organizations.* American Sociological Review, 1976. **41**(2): p. 322-338.
38. Howarth, E., K. Devers, G. Moore, et al., *Contextual issues and qualitative research*, in *Challenges, solutions and future directions in the evaluation of service innovations in health care and public health*. 2016, NIHR Journals Library.
39. Craig, P., P. Dieppe, S. Macintyre, et al., *Developing and evaluating complex interventions: the new Medical Research Council guidance.* Bmj, 2008. **337**: p. a1655.

For peer review only - http://bmjopen.bmj.com/site/about/guidelines.xhtml

40. Sheetz, K.H. and M.J. Englesbe, *Expanding the Quality Collaborative Model as a Blueprint for Higher-Value Care.* JAMA Health Forum, 2020. **1**(5): p. e200413-e200413.
41. Dixon-Woods, M., G. Martin, C. Tarrant, et al., *Safer Clinical Systems: evaluation findings*. 2014: London.
42. Dixon-Woods, M., S. McNicol, and G. Martin, *Ten challenges in improving quality in healthcare: lessons from the Health Foundation's programme evaluations and relevant literature.* BMJ Quality & Safety, 2012. **21**(10): p. 876.
43. Woodcock, T., E.G. Liberati, and M. Dixon-Woods, *A mixed-methods study of challenges experienced by clinical teams in measuring improvement.* BMJ Quality & Safety, 2021. **30**(2): p. 106-115.
44. Needham, D.M., D.J. Sinopoli, V.D. Dinglas, et al., *Improving data quality control in quality improvement projects.* International Journal for Quality in Health Care, 2009. **21**(2): p. 145-150.
45. Pronovost, P.J., T. Nolan, S. Zeger, et al., *How can clinicians measure safety and quality in acute care?* The Lancet, 2004. **363**(9414): p. 1061-1067.
46. Ghaferi, A.A. and J.B. Dimick, *Practical Guide to Surgical Data Sets: Medicare Claims Data.* JAMA Surgery, 2018. **153**(7): p. 677-678.
47. Lawson, E.H., R. Louie, D.S. Zingmond, et al., *A Comparison of Clinical Registry Versus Administrative Claims Data for Reporting of 30-Day Surgical Complications.* Annals of Surgery, 2012. **256**(6): p. 973-981.
48. Osborne, N.H., L.H. Nicholas, A.M. Ryan, et al., *Association of Hospital Participation in a Quality Reporting Program With Surgical Outcomes and Expenditures for Medicare Beneficiaries.* JAMA, 2015. **313**(5): p. 496-504.
49. Ling, T., *How do you get clinicians involved in quality improvement?: an evaluation of the Health Foundation's engaging with quality initiative: a programme of work to support clinicians to drive forward quality*. 2010: The Health Foundation.
50. Benning, A., M. Ghaleb, A. Suokas, et al., *Large scale organisational intervention to improve patient safety in four UK hospitals: mixed method evaluation.* Bmj, 2011. **342**: p. d195.
51. Benn, J., S. Burnett, A. Parand, et al., *The journey to safety: a report of 24 NHS organisations undertaking the Safer Patients Initiative.* 2011.
52. Benn, J., S. Burnett, A. Parand, et al., *Studying large-scale programmes to improve patient safety in whole care systems: Challenges for research.* Social Science & Medicine, 2009. **69**(12): p. 1767-1776.
53. Dixon-Woods, M. and G.P. Martin, *Does quality improvement improve quality?* Future Hosp J, 2016. **3**(3): p. 191-194.
54. Mountford, J. and K.G. Shojania, *Refocusing quality measurement to best support quality improvement: local ownership of quality measurement by clinicians.* BMJ Quality & Safety, 2012. **21**(6): p. 519-523.
55. Dixon-Woods, M., M. Leslie, J. Bion, et al., *What Counts? An Ethnographic Study of Infection Data Reported to a Patient Safety Program.* The Milbank Quarterly, 2012. **90**(3): p. 548-591.
56. Dixon-Woods, M., A. Campbell, E.-L. Aveling, et al., *An ethnographic study of improving data collection and completeness in large-scale data exercises.* Wellcome open research, 2019. **4**: p. 203-203.
57. Campbell, D.A., *Quality Improvement Is Local.* Journal of the American College of Surgeons, 2009. **209**(1): p. 141-143.
58. Health Foundation, *Learning report: Safer Patients Initiative. Lessons from the first major improvement programme addressing patient safety in the UK.* 2011.
59. Bardsley, M., *Understanding analytical capability in health care. Do we have more data than insight?* 2016, Health Foundation: London.
60. Lillo-Crespo, M., M.C. Sierras-Davó, A. Taylor, et al., *Mapping the Status of Healthcare Improvement Science through a Narrative Review in Six European Countries.*

For peer review only

For peer review only - http://bmjopen.bmj.com/site/about/guidelines.xhtml

International Journal of Environmental Research and Public Health, 2019. **16**(22): p. 4480.
61. Wright, D., J. Gabbay, and A. Le May, *Determining the skills needed by frontline NHS staff to deliver quality improvement: findings from six case studies.* BMJ Quality & Safety, 2022. **31**(6): p. 450-461.
62. Martin, G.P., *Beyond improvement skills: what do clinicians, managers, patients and others need to do to make improvement happen?* BMJ Quality & Safety, 2022. **31**(6): p. 423-425.
63. Young, G.J., *Multistakeholder Regional Collaboratives Have Been Key Drivers Of Public Reporting, But Now Face Challenges.* Health Affairs, 2012. **31**(3): p. 578-84.
64. Krapohl, G.L., M.R. Hemmila, S. Hendren, et al., *Building, scaling, and sustaining a learning health system for surgical quality improvement: A toolkit.* Learning Health Systems, 2020. **4**(3): p. e10215.
65. Ivers, N., G. Jamtvedt, S. Flottorp, et al., *Audit and feedback: effects on professional practice and healthcare outcomes.* Cochrane Database of Systematic Reviews, 2012(6).
66. Dimick, J.B. and S.K. Hendren, *Hospital Report Cards: Necessary but Not Sufficient?* JAMA Surgery, 2014. **149**(2): p. 143-144.
67. Glance, L.G., T.M. Osler, D.B. Mukamel, et al., *Effectiveness of Nonpublic Report Cards for Reducing Trauma Mortality.* JAMA Surgery, 2014. **149**(2): p. 137-143.
68. Prang, K.-H., R. Maritz, H. Sabanovic, et al., *Mechanisms and impact of public reporting on physicians and hospitals' performance: A systematic review (2000–2020).* PLOS ONE, 2021. **16**(2): p. e0247297.
69. Campanella, P., V. Vukovic, P. Parente, et al., *The impact of Public Reporting on clinical outcomes: a systematic review and meta-analysis.* BMC Health Services Research, 2016. **16**(1): p. 296.
70. Carter, P., P. Ozieranski, S. McNicol, et al., *How collaborative are quality improvement collaboratives: a qualitative study in stroke care.* Implementation Science, 2014. **9**(1): p. 32.
71. Freidson, E., *Professionalism, the third logic: On the practice of knowledge*. 2001: University of Chicago press.
72. Martin, G.P., N. Armstrong, E.-L. Aveling, et al., *Professionalism Redundant, Reshaped, or Reinvigorated? Realizing the "Third Logic" in Contemporary Health Care.* Journal of Health and Social Behavior, 2015. **56**(3): p. 378-397.
73. Evetts, J., *New Professionalism and New Public Management: Changes, Continuities and Consequences.* Comparative Sociology, 2009. **8**(2): p. 247-266.
74. Dückers, M.L.A., C. Wagner, L. Vos, et al., *Understanding organisational development, sustainability, and diffusion of innovations within hospitals participating in a multilevel quality collaborative.* Implementation Science, 2011. **6**(1): p. 18.
75. Parand, A., J. Benn, S. Burnett, et al., *Strategies for sustaining a quality improvement collaborative and its patient safety gains.* International Journal for Quality in Health Care, 2012. **24**(4): p. 380-390.
76. Dixon-Woods, M., C.L. Bosk, E.-L. Aveling, et al., *Explaining Michigan: Developing an Ex Post Theory of a Quality Improvement Program.* The Milbank Quarterly, 2011. **89**(2): p. 167-205.
77. Bovens, M., *Analysing and assessing accountability: A conceptual framework.* European Law Journal, 2007. **13**(4): p. 447-468.
78. Bovens, M., T. Schillemans, and P.t. Hart, *Does public accountability work? An assessment tool.* Public Administration, 2008. **86**(1): p. 225-242.

For peer review only - http://bmjopen.bmj.com/site/about/guidelines.xhtml

A qualitative study of the Michigan collaborative quality initiatives

Interview topic guide

20.08.2017

Study aims

1. Produce a thorough empirical description of what it is that the Michigan CQIs do: the activities they undertake, the structures through which they operate, and the resources they use, for example.
2. Identify the program theory of the Michigan CQIs, including identification of specific interventions and their contextual influences, in so doing characterising the mechanisms of their success.
3. Generate learning about the distinctive features of the Michigan approach for generalisability and reproduction of improvement in other settings.

Interviewees

This document presents a topic guide for interviews with personnel who have participated or are actively participating in one or more of the Michigan collaborative quality initiatives (CQIs). Relevant personnel may include (but are not limited to):

- Senior leaders from Blue Cross Blue Shield of Michigan.
- Leaders of the CQIs.
- Program managers and implementers of CQI quality improvement programs.
- Front line staff from Michigan hospitals who participate in the CQIs.

Questions used may vary by role of personnel in the CQI, i.e. not all questions may be relevant for every interviewee.

Topic structure

1. Questions about role and relevant CQI(s).
2. Questions to elicit empirical description.

For peer review only

A qualitative study of the Michigan collaborative quality initiatives

Interview topic guide

20.08.2017

3. Questions to identify program theory.
4. Questions to identify features of context.
5. Questions about generalizability.
6. Safety net.

1. Questions about role and relevant CQI

1. Which CQI(s) were you involved in the past, or are you involved in now?

2. What was/is your role in the CQI(s)?

3. Please tell me about your role in the CQI(s).

2. Questions to elicit empirical description

Aim: Produce a thorough empirical description of what it is that the Michigan CQIs do: the activities they undertake, the structures through which they operate, and the resources they use, for example.

Structures, processes and activities

4. Please describe how the collaborative works – how is it structured, and what are its key processes and activities?

5. How do you ensure key processes and activities happen effectively and in standardized or harmonized way across different sites?

Resources

6. What resources does the collaborative need to operate? Which resources are particularly important, and why?

For peer review only

A qualitative study of the Michigan collaborative quality initiatives

Interview topic guide

20.08.2017

Topic selection and prioritization

7. How are topics or problems for quality improvement selected and prioritized? How are these decisions taken and by whom? How far is there consensus on what is important?

Definition and identification of positive deviance

8. How are high performing hospitals defined and identified? Are you most interested in hospital, unit or individual clinician performance? Why?

Technical components and interventions

9. What are the technical components, e.g. information technology and communications infrastructure, needed for improvement to happen? What kind of testing did you do to figure out which components were essential? Were there any challenges in making sure all components worked reliably at different sites?

10. How does technology enable improvement in Michigan? How is new technology selected and tested?

11. When variation in quality or practice is identified, how are best practices at the high performing hospitals identified?

12. Among the identified 'best practices' of high performing hospitals, how are the most important interventions to improve quality at other hospitals identified and selected?

13. How are these best practices developed and tested as quality improvement interventions, and how are they then introduced at other sites? What are the important processes and activities involved in this? How does it happen in practice?

For peer review only

A qualitative study of the Michigan collaborative quality initiatives

Interview topic guide

20.08.2017

Technical and operational expertise

14. What technical expertise is required for the collaborative to operate effectively? Is training required? Who receives training and how does that happen?

15. Who are the key people responsible for ensuring the collaborative works effectively on a day-to-day basis? Are the systems they use to achieve this reliable? What are they?

Data

16. What data are most relevant for the purposes of improving quality of care? Why? What process was used to prioritise data and what role did clinicians play in this?

17. How has data and its collection been standardized between participating hospitals? How did that process happen in practice?

18. How are data collected in Michigan?

19. Who is responsible for collecting data? What role do participating hospitals, units and clinicians have in the collection and preparation of data?

20. Please tell me about the data infrastructure used in Michigan. How does it provide what you need to improve quality? What are its most important features?

21. Was the data infrastructure planned and tested before the collaborative launched? Did you have a clear idea about what you needed in advance? How has it changed over time and why did it change?

For peer review only

A qualitative study of the Michigan collaborative quality initiatives

Interview topic guide

20.08.2017

22. How does the collaborative assure the quality of its data?

23. How much credibility does data have among participants? Where does that credibility come from? Why do participants trust the data?

24. Please tell me about how data is used to improve quality in Michigan. What are the key processes involved?

25. In your experience, what is unique or different about how data is collected and used in Michigan?

26. Is data used to rank or compare participants in any way? Does this happen at the level of hospitals, units or individuals? Can sites or individuals compare their performance?

Feedback

27. What information is fed back to participants? What aspects of quality or performance are highlighted as most important and why, e.g. are outcomes more important than process measures? How was this determined and what role did clinicians play?

28. How is information fed back to participants? What are the key processes and activities? To whom and how frequently does feedback occur? At what levels are information presented, e.g. hospital, unit, individual?

29. In what format is information fed back, e.g. use of visuals, text, numerical? How was this format developed? Who was involved in the process of developing this format? What role did clinicians play in developing it?

30. To what extent is information customised for individuals? Does the information presented vary by type of personnel, e.g. between doctors and nurses?

For peer review only

A qualitative study of the Michigan collaborative quality initiatives

Interview topic guide

20.08.2017

31. How is information fed back in a way that makes it actionable for improvement? How and at what level are actions identified, e.g. for hospitals or individual clinicians? How are action plans developed?

32. Is the approach to feedback in Michigan based on any particular theories? Was a particular approach to feedback planned from the beginning?

33. In your experience, what is unique or different about how feedback happens in Michigan compared to elsewhere?

Communication

34. What are the important communication principles and processes? What makes communication in Michigan effective?

35. Is there direct communication between participants in the collaborative? How does this happen in practice?

Research outputs

36. Why are the collaboratives able to publish so many of their results as research papers? What infrastructure and relationships are in place to support this approach? How does it happen in practice?

3. Questions to identify program theory

Aim: Identify the program theory of the Michigan CQIs, including identification of specific interventions and their contextual influences, in so doing characterising the mechanisms of their success.

For peer review only

A qualitative study of the Michigan collaborative quality initiatives

Interview topic guide

20.08.2017

Aims and development

37. Can you tell me what your original aim was? What problem was the collaborative created to solve? Did the aim change over time?

38. In the beginning, how was it imagined that the collaborative would achieve that aim? What structures and processes were assumed to be important, and why? By what mechanisms were these intended to improve quality?

39. Can you tell me how the collaborative was originally developed, and why it was developed in that way? What was the role of research evidence?

40. Over time, did the way you sought to improve quality change? What changed and why?

Key interventions for improvement

41. Can you give some examples of important interventions that make improvement happen in Michigan? How do they work?

42. Do any of these interventions work differently in practice to what was anticipated? Are some interventions more important or less important than you anticipated?

43. Are there any structures, processes or activities that developed and became important for improvement, that were not initially imagined or planned?

44. In your experience, what advantages does the Michigan approach have over other methods improving quality? What is different about what happens in Michigan that makes you better at improving quality?

For peer review only

8

A qualitative study of the Michigan collaborative quality initiatives

Interview topic guide

20.08.2017

Learning for improvement

45. How do you know if you are achieving improvement, and how do you know if that improvement is attributable to your interventions?

46. How do hospitals learn from each other in Michigan? How is learning maximised?

47. How is learning used to improve quality? How does this happen in practice?

48. What are the key features of the Michigan approach that ensure learning is captured and retained?

49. How does the collaborative build organizational memory, and how is it accessed by participants over time?

50. Are there any components of the collaborative you think might be less effective? If you had to drop anything, what would it be, and why?

Academic approach

51. The collaboratives publish a lot of their results as research papers. Was this a planned feature of the collaborative from the outset? Was this a rationale/incentive for setting up the collaborative in the first place? Why?

52. What role does this academic approach play in conferring legitimacy for the collaborative's purpose and activities? Does the importance of this vary between participants, e.g. between program leaders and front-line clinicians? How does it vary?

53. What role do research outputs play in creating and sustaining engagement and motivation among clinicians?

For peer review only

A qualitative study of the Michigan collaborative quality initiatives

Interview topic guide

20.08.2017

Role of competition in improvement

54. Does a sense of competition – for example between hospitals -- have a role in improving quality in Michigan? Why or why not?

55. Do sites or individuals involved in the collaborative feel like they are in competition with each other? Why or why not?

56. At what level is competition most relevant or keenly felt, e.g. between hospitals, between units, between individuals?

Role of collaboration in improvement

57. What role does direct collaboration between participating sites or clinicians play in improvement?

Role of innovation in improvement

58. What is the role of innovation in how hospitals improve? Is innovation an important part of how improvement happens? What is distinctive about innovation in Michigan?

4. Questions to identify features of context

Aim: Identify the program theory of the Michigan CQIs, including identification of specific interventions and their contextual influences, in so doing characterising the mechanisms of their success.

For peer review only

10

A qualitative study of the Michigan collaborative quality initiatives

Interview topic guide

20.08.2017

General contextual influences

59. What factors do you think make your approach to improvement more or less likely to work?

60. What factors influence the effectiveness of the interventions for improvement?

61. Thinking about the factors which influence success, what do you think are the minimum prerequisites for a collaborative approach to improving quality to work?

Political and policy environment

62. What factors in the political or policy environment in Michigan affect how improvement happens?

63. Have political or policy factors changed over time? How has that impacted improvement?

64. Please tell me about the relationship between the insurer (BCBSM) and the collaborative.

Culture and social environment

65. Please tell me about the culture and ethos in Michigan and in your collaborative in particular. What are they, where do they come from and how are they sustained? What are the cultural hallmarks of the Michigan approach to improvement? To what extent are they shared within the community of participants?

66. How do partners in the collaborative build trust?

For peer review only

A qualitative study of the Michigan collaborative quality initiatives

Interview topic guide

20.08.2017

Leadership

67. What role does leadership play in improvement in Michigan? How important is clinical leadership in particular?

68. How is the collaborative led? How are the culture and ethos of the collaborative reflected in how it is led?

Clinical engagement

69. In the early days, how were clinicians persuaded to engage with a new approach to improvement?

70. Did the methods used to engage clinicians change over time?

71. Are the factors important in achieving and sustaining clinical engagement different? What keeps clinicians engaged and motivated once they are 'bought in'?

72. How are clinicians persuaded and motivated to change practice, and adopt the practice of other hospitals or clinicians? How is that loss of autonomy managed?

73. Are any hospitals or clinicians disengaged? Why?

Innovation

74. How is a supportive environment for innovation created in Michigan?

75. What features of the environment are important to enable innovation?

For peer review only

A qualitative study of the Michigan collaborative quality initiatives

Interview topic guide

20.08.2017

5. Questions about generalizability

Aim: Generate learning about the distinctive features of the Michigan approach for generalizability and reproduction of improvement in other settings.

76. Would the Michigan approach work anywhere? Why or why not?

77. What advice would you give to other healthcare organizations thinking about adopting a collaborative approach to quality improvement?

78. Would your advice change if that organization was in a different healthcare system, such as the UK?

79. What do think are the most important features of the Michigan approach to improvement, without which it would be impossible to reproduce its success elsewhere?

80. In your experience, what were the most important challenges to improving quality and how were they overcome?

81. What is missing from the Michigan approach that could improve it further? How would you like to see it develop in the future?

6. Safety net

For peer review only

A qualitative study of the Michigan collaborative quality initiatives

Interview topic guide

20.08.2017

82. Is there something else about how and why quality and safety have improved in Michigan you would like to add?

For peer review only

Supplementary Table 1~~2~~. Summary of distinctive features of the CQIs programme, associated ~~themes~~data categories and examples of supporting data

Feature	~~Theme~~Data categories	~~Supporting~~Illustrative supporting data
**Learning from positive deviance**	*Making knowledge accessible to support improvement*	*~~So when~~When you identify the outliers as high performers, you have an opportunity to ask them, what is your technique, for whether it's a procedure technique or a follow-up technique or getting your patients to come back for further data points down the road, you have an opportunity to learn from them ~~…(T18~~(Surgeon)*
	Learning from the collaborative community	*So for example, Flint, Michigan is a town with a lot of challenges, but one of the two big hospitals there provided the best acute care surgery in the state. So ~~it's like, oh,~~ that is a reason to celebrate and to have them, [a] high performer, teach the rest of us~~.(T8~~. (Surgeon)*
**High-quality coordination**	*Offloading administrative and technical burdens from local ~~sites~~hospitals and clinicians*	*We do the data analysis for them…So, they'll send in their data and we'll make it pretty for them… So, we just don't shove data to them and say, 'hey, do the best you can, but you've got to improve.' We actually provide face~~-~~-to~~-~~-face quality training. We will go into the practice and do mentorship. We'll go into the practice and consult on the practice, if they're really struggling. And then we provide*

For peer review only

		resources for them, so that they don't have to start with white pieces of paper, to kind of initiate their quality improvement project. So, we give them a lot of tools [to] help both with engagement, but success. (T20(Manager)<br><br>*They [the CQI coordinating centre] will assist you in analysing your data from your baseline reporting period, and say you have a higher utilisation, length of stay, ED visit or readmission rate than the rest of the collaborative, [the coordinating centre] would encourage you to focus on these things. (Nurse)*
	Coordinating knowledge exchange	*And then weWe are constantly hooking sites up, so today I'm trying to hook, for example, a site up who is doing a certain procedure for Medicare patients. They're having a hard time getting it approved so I went in the database and I looked through to see, okay, who else is doing this same procedure for this insurance population, okay, so then I reached out to that coordinator and said, "hey, we have a site who's having a hard time. Would you mind sharing anything that's been helpful??" So we do a lot of this.(T17. (Nurse)*

**High-quality measurement and comparative performance feedback**	*Building confidence in data through clinical ownership and standardisation*	*These collaboratives are developed by the physicians, and by far ..it's their own data, so they believe it. (T31Manager)*<br><br>*My preferred data to share is how we compare to the other hospitals. And my confidence in doing that is higher with that particular data because whether I'm an abstractor in a Detroit hospital or in a hospital in northern Michigan, we still have to follow the same definitions, abstract the same way. And once the surgeons understood that they did not question the validity of my data as much. (Nurse)*
	Identifying improvement opportunities	*Well, if you don't know how you're performing compared to seventy-two other hospitals, how can you effect change? (T5Surgeon)*<br><br>*If you have an increase or a high number of SSIs [surgical site infections], then they [the CQI coordinating centre] will recommend that you focus on reducing surgical site infections, and these are some of the ways that you can do it. So not everybody does the same thing, it depends on where they're lacking. (Nurse)*
	Creating accountability for quality	*..twoTwo surgeons go, [to the service being audited], we send them [the audited service] a formal report back and then they have to stand up*

For peer review only - http://bmjopen.bmj.com/site/about/guidelines.xhtml

		at the next collaborative ~~or the next one, so~~[meeting] within three-to-six months [to] show us their performance improvement plan and where they are~~.(T23~~. (Surgeon)
**Careful use of motivational levers**	*Motivating participants to improve*	*What I would say in terms of motivation is there's a lot of motivational levers and that part of running the CQI is that no one lever works for every single person. (~~T1~~Surgeon)*
	Safeguards to preserve healthy competition	*~~..part~~Part of the trust that we've been able to establish, and we have emphasised over and over again that Blue Cross, Blue Shield of Michigan will never see their data. (~~T19~~Manager)*
**Mobilising professional leadership and building community**	*Building relationships and community*	*~~Really important because fundamentally~~Fundamentally, they get to see each other and they actually become a community of care givers who know each other and interact, have enough interactions with each other that it really strengthens~~, essentially,~~ the coherence of the delivery system~~.(T10~~. (Manager)*
	Preserving an environment of trust	*They always start their meeting with ~~kind of~~ a general statement, like a confidentiality ~~kind of like~~ statement in the presentation, ~~like,~~ you're all here, we didn't ask you to sign it, but we expect you to ~~kind of~~ adhere to this understanding~~.(T12~~. (Manager)*

	Preserving professional autonomy	*The fact that they [clinicians] are the ones doing their own interventions to try to make things better, I also consider a success factor of this. They're not told, you've got to use X model to be able to improve this. They are given, I think, complete autonomy as to how they make the improvements in their own area.(T29. (Manager)*

~~Source: Authors' analysis of case study data~~

For peer review only

Standards for Reporting Qualitative Research (SRQR)

Manuscript title: What are the features of high-performing quality improvement collaboratives? Qualitative case study of a state-wide collaboratives programme

Title and abstract

	Addressed	Page(s) (unmarked manuscript)
**Title -** Concise description of the nature and topic of the study Identifying the study as qualitative or indicating the approach (e.g., ethnography, grounded theory) or data collection methods (e.g., interview, focus group) is recommended	Yes	1
**Abstract -** Summary of key elements of the study using the abstract format of the intended publication; typically includes background, purpose, methods, results, and conclusions	Yes	2

Introduction

**Problem formulation -** Description and significance of the problem/phenomenon studied; review of relevant theory and empirical work; problem statement	Yes	4-5
**Purpose or research question -** Purpose of the study and specific objectives or questions	Yes	4-5

Methods

**Qualitative approach and research paradigm -** Qualitative approach (e.g., ethnography, grounded theory, case study, phenomenology, narrative research) and guiding theory if appropriate; identifying the research paradigm (e.g., postpositivist, constructivist/ interpretivist) is also recommended; rationale**	Yes	5-7
**Researcher characteristics and reflexivity -** Researchers' characteristics that may influence the research, including personal attributes, qualifications/experience, relationship with participants, assumptions, and/or presuppositions; potential or actual interaction between researchers' characteristics and the research questions, approach, methods, results, and/or transferability	No	See cover letter
**Context -** Setting/site and salient contextual factors; rationale**	Yes	5-7
**Sampling strategy -** How and why research participants, documents, or events were selected; criteria for deciding when no further sampling was necessary (e.g., sampling saturation); rationale**	Yes	5-7
**Ethical issues pertaining to human subjects -** Documentation of approval by an appropriate ethics review board and participant consent, or explanation for lack thereof; other confidentiality and data security issues	Yes	5-7

For peer review only - http://bmjopen.bmj.com/site/about/guidelines.xhtml

**Data collection methods -** Types of data collected; details of data collection procedures including (as appropriate) start and stop dates of data collection and analysis, iterative process, triangulation of sources/methods, and modification of procedures in response to evolving study findings; rationale**	Yes	5-7
**Data collection instruments and technologies -** Description of instruments (e.g., interview guides, questionnaires) and devices (e.g., audio recorders) used for data collection; if/how the instrument(s) changed over the course of the study	Yes	5-7
**Units of study -** Number and relevant characteristics of participants, documents, or events included in the study; level of participation (could be reported in results)	Yes	5-9
**Data processing -** Methods for processing data prior to and during analysis, including transcription, data entry, data management and security, verification of data integrity, data coding, and anonymization/de-identification of excerpts	Yes	5-7
**Data analysis -** Process by which inferences, themes, etc., were identified and developed, including the researchers involved in data analysis; usually references a specific paradigm or approach; rationale**	Yes	5-7
**Techniques to enhance trustworthiness -** Techniques to enhance trustworthiness and credibility of data analysis (e.g., member checking, audit trail, triangulation); rationale**	Yes	5-7

Results/findings

**Synthesis and interpretation -** Main findings (e.g., interpretations, inferences, and themes); might include development of a theory or model, or integration with prior research or theory	Yes	8-14
**Links to empirical data -** Evidence (e.g., quotes, field notes, text excerpts, photographs) to substantiate analytic findings	Yes	8-14

Discussion

**Integration with prior work, implications, transferability, and contribution(s) to the field -** Short summary of main findings; explanation of how findings and conclusions connect to, support, elaborate on, or challenge conclusions of earlier scholarship; discussion of scope of application/generalizability; identification of unique contribution(s) to scholarship in a discipline or field	Yes	14-17
**Limitations -** Trustworthiness and limitations of findings	Yes	3 & 14-17

Other

**Conflicts of interest -** Potential sources of influence or perceived influence on study conduct and conclusions; how these were managed	Yes	18
**Funding -** Sources of funding and other support; role of funders in data collection, interpretation, and reporting	Yes	18

For peer review only
